# Comparative analysis of xenogeneic collagen matrix and autogenous subepithelial connective tissue graft to increase soft tissue volume around dental implants: a systematic review and meta-analysis

**DOI:** 10.1186/s12903-023-03475-0

**Published:** 2023-10-10

**Authors:** Igor Ashurko, Svetlana Tarasenko, Mary Magdalyanova, Svetlana Bokareva, Maxim Balyasin, Anna Galyas, Marina Khamidova, Mariia Zhornik, Alexey Unkovskiy

**Affiliations:** 1 Department of Dental Surgery, Sechenov First Moscow State Medical University, Bolshaya Pirogovskaya Street, 19c1, Moscow, 119146 Russia; 2 Peoples’ Friendship University, Moscow, Russia; 3 Department of Prosthodontics, Geriatric Dentistry and Craniomandibular Disorders, Charité -Universitätsmedizin Berlin, Corporate Member of Freie Universität Berlin, Humboldt-Universität Zu Berlin, Aßmannshauser Str. 4-6, 14197 Berlin, Germany

**Keywords:** Dental implant, Connective tissue graft, Subepithelial connective tissue graft, Soft tissue, Collagen matrix, Soft tissue augmentation, Thickness increasing, Systematic review

## Abstract

**Objective:**

The gold standard for a soft tissue augmentation around implants is a subepithelial connective tissue graft (CTG), but the xenogeneic collagen matrices (XCM) started to be used as an alternative. This systematic review aimed to assess the effectiveness XCM in comparison to CTG for the increasing the thickness of the soft tissue around implants.

**Data:**

All studies included at least two parallel groups comparing the use of CTG and XCM with a minimum follow-up of 3 months. As the primary outcome, the amount of soft tissue thickness gain after soft tissue augmentation with XCM or CTG was assessed. Secondary outcomes were clinical and patient-related outcomes; evaluation of aesthetic outcomes, patient-reported outcomes measures (PROMs) and complications. Eligible studies were selected based on the inclusion criteria. Meta-analysis was applied whenever possible. The quality of the evidence of studies including in meta-analysis was assessed using the GRADE approach.

**Source:**

A systematic literature search up to January 2022 was conducted using the following electronic databases: PubMed (MEDLINE), Scopus, Cochrane Library, LILACS, eLIBRARY.RU. Unpublished researches, the gray literature, nonprofit reports, government studies and other materials were reviewed electronically using an EASY search. An additional manual search was carried out in November 2022.

**Study selection:**

Of the 1376 articles from the initial search, 8 randomized controlled trials (RCTs) (306 patients and 325 implants) were included in this systematic review, and 7 studies were part of the meta-analysis. Meta-analysis revealed that XCM is less effective than the CTG in increasing soft tissue thickness around dental implants. However, XCM also provides soft tissue thickness gain and can be recommended for use in various clinical situations.

**Clinical significance:**

Previous systematic reviews and meta-analyses have shown that autologous grafts are more effective than collagen matrices in increasing soft tissue thickness, however, the latter can be used as an alternative. Studies included in previous systematic reviews varied in design, which could lead to limitations. The present systematic review and meta-analysis includes for the first time only randomized controlled clinical trials with collagen matrix of xenogeneic origin in the test group. Tight eligibility criteria were established, and the main parameter studied was soft tissue thickness. It was found that xenogeneic collagen matrix is effective for increasing soft tissue thickness around dental implants, however, the results obtained using an autogenous connective tissue graft are superior.

## Introduction

Nowadays, the use of dental implants can be regarded as widespread and predictable procedure for the replacement of missing teeth [1]. The presence of sufficient amount of bone and soft tissues surrounding dental implants allows attaining long-term outcomes of implant treatment [2, 3]. An adequate keratinized mucosa width (KMW) of ≥ 2 mm around dental implants refers to crucial factor preventing inflammatory complications and unsatisfactory esthetic result [4, 5].

It has been reported that soft tissue thickness is also regarded as decisive volumetric parameter affecting peri-implant marginal bone level. In 1996, Berglundh and Lindhe revealed that a minimum of 2 mm gingival thickness is required for establishment of biological width around implants, and if the thickness is insufficient, bone loss will occur [6]. These findings were further confirmed by Linkevicius et al. [7, 8]. It should also be noted that the thin mucosal tissue in the area of the implants might have a grayish color due to the visibility of the abutment [9].

In recent times the topic of peri-implant soft tissue management gains increasingly more interest in the scientific literature. Currently, autogenous connective tissue graft (CTG) taken from the palate or maxillary tuberosity is represented as the gold standard for soft tissue thickening [10]. However, such disadvantages of harvesting procedure as complexity and duration of manipulation, limitations in graft size, as well as the possibility of prolonged bleeding, infection risk and postoperative discomfort at the donor site have been reported [11, 12].

In order to simplify the clinical procedure, the acellular dermal matrix, amniotic membrane of human origin, as well as a range of synthetic materials can be alternatively applied [13, 14]. The efficacy of xenogeneic collagen matrices (XCM) has been also described, demonstrating volume stability over a period of time, good tissue integration, promotion of new blood vessels formation and cellular ingrowth [15, 16].

The main representatives of XCMs are bilayered collagen matrix (CM), volume-stable collagen matrix (VCMX), acellular dermal matrix (ADM) and extracellular matrix. Each of them has certain advantages, for instance, the bilayered CM has an excellent color match with the surrounding tissue, while maintaining stability and elasticity distinguishes the VCMX from others. The application of ADM contributes to enhanced proliferation of fibroblasts, endothelial cells and tissue revascularization, while extracellular matrix is also capable to stimulate cell adhesion, differentiation, and vascular ingrowth, which aids a more predictable outcome [14].

Recent studies have been focused on comparative analysis between XCMs and CTGs in terms of their aid to the quality and quantity of soft tissues around dental implants after soft tissue augmentation [17–19]. Investigations were aimed to assess changes in soft tissue dimensions, periodontal status, and patient satisfaction after augmentation by XCM or CTG [20]. The leading volumetric parameter that was given attention in previous systematic reviews was KMW, while data on changes in soft tissue thickness were often of secondary importance [12, 21]. Whereas there have been a sufficient variety of studies to the soft tissue management, their results remain controversial with a low number of randomized clinical trials, resulting in a poor evidence to the present topic. Consequently, the present systematic review and meta-analysis aimed to evaluate the effectiveness of XCM in comparison to CTG for increasing the soft tissue thickness around dental implants.

## Materials and methods

### Study registration and protocol development

The protocol of the present review was registered and allocated the identification number CRD42022297500 in the International Prospective Register of Systematic Reviews (PROSPERO). This manuscript was prepared following the Cochrane Collaboration guidelines [22] and reported in accordance to the standards of the Preferred Reporting Items for Systematic Reviews and Meta Analyses (PRISMA) Statement [23].

### Objectives

The goal of this review was to analyze and compare effectiveness of modern XCM and autogenous subepithelial CTG in increasing soft tissue thickness around dental implants.

### PICOT Question and Focused Question

*Population (P)*: patients who underwent soft tissue grafting (XCM, CTG) under the full or split-thickness flap to increase the thickness (volume) of soft tissues in the area of dental implants (during implant placement or during the healing abutment placement) for aesthetic, biological and functional reasons.

*Intervention (I)*: surgical intervention that was performed by placing the graft (XCM) in the implant area under the full or split-thickness flap.

*Comparison (C)*: in the control group the operation was performed with the use of CTG. The grafts from different donor areas are included in the analysis: CTG or subepithelial connective tissue graft (SCTG) harvested from the palate or maxillary tuberosity, de-epithelized free gingival graft (FGG).

*Outcome (O)*: the main outcome was to evaluate increasing soft tissue thickness (volume) in the area of implants, additional outcomes included changes in clinical and radiographic peri-implant outcomes (width of keratinized mucosa, plaque index, bleeding indices, probing depth, marginal bone levels, surgery time), professional evaluation of aesthetic outcomes (pink and white esthetic scores), PROMs – pain syndrome, quality of life and complications.

*Time (T)*: Minimum follow-up of 3 months after the surgical intervention.

The focused question is: Can modern XCMs provide results comparable to autogenous CTGs in increasing soft tissue thickness in the area of dental implants?

### Eligibility criteria

#### Inclusion criteria

- XCM or CTG augmentation at implant site
- Prospective randomized controlled interventional human studies
- Evaluation and reporting of clinical outcomes of increasing soft tissue thickness in the implant area using XCM and/or autogenous CTG over a minimum follow-up period of 3 months.

#### Exclusion criteria

- Retrospective and cross-sectional clinical studies, reviews, case reports or animal studies
- Increasing solely the KMW in the area of the implants
- Application of FGG, allogenic and alloplastic (synthetic) materials for soft tissue augmentation
- Increasing soft tissue volume or thickness around natural teeth
- Soft tissue augmentation with immediate implant placement into the socket of the extracted tooth
- Augmentation of soft tissues simultaneously with bone grafting

### Information sources and search strategy

#### Selection of studies

A detailed systematic literature search was conducted by three researchers (IA, MZ and MM) using the following electronic databases: The National Library of Medicine (MEDLINE via PubMed); Scopus, the Cochrane Library, Latin American & Caribbean Health Sciences Literature (LILACS), eLIBRARY.ru. For examining unpublished trials, the grey literature, nonprofit reports, government research or other materials were also electronically explored through searching in EASY.

The search strategy was primarily designed for the PubMed (MEDLINE) database with advanced search and free text terms, and then adapted accordingly the other databases. There were no restrictions on publication date, language or journal in all databases, except for the LILACS database, where a language restriction was applied (only articles in English). The search was conducted on January 5, 2022. The PubMed (MEDLINE) search was rerun in July 2022. Also in November 2022, an additional manual search was carried out. Details regarding the search strategy are reported in Table 1. In addition, reference lists of relevant studies and full-texts of previous systematic reviews investigating soft tissue management around dental implants were also screened [10, 12, 17, 21, 24]. The search results were downloaded to a Rayyan QCRI (Qatar Computing Research Institute, Doha, Qatar) to facilitate duplicate removal and cross-reference checks.

**Table 1:** Electronic databases and search strategies

Databases	Keywords
PubMed	#1 "collagen matrix" OR "collagen matrices" OR "collagen-based matrix" OR "mucograft" OR "mucoderm" OR "xenogeneic graft material" OR "acellular dermal matrix" OR "extracellular membrane" OR "extracellular matrix" OR "collagen" [MeSH Terms] #2 "soft tissue augmentation" OR "soft tissue grafting" OR "soft tissue volume" OR "soft tissue thickness" OR "soft tissue thickening" OR "guided tissue regeneration" OR "soft tissue management" OR "soft tissue correction" OR "connective tissue graft" OR "connective tissue"[MeSH Terms] OR "autogenous graft" OR "soft tissue graft" #3 "dental implants"[MeSH Terms] OR "dental implantation"[MeSH Terms] OR "dental implant*" #1 AND #2 AND #3
Scopus	#1 TITLE-ABS-KEY("collagen matrix" OR "collagen matrices" OR "collagen-based matrix" OR "mucograft" OR "mucoderm" OR "fibrogide" OR "xenogeneic graft material" OR "acellular dermal matrix" OR "extracellular membrane" OR "extracellular matrix") #2 TITLE-ABS-KEY("soft tissue augmentation" OR "soft tissue grafting" OR "soft tissue volume" OR "soft tissue thickness" OR "soft tissue thickening" OR "guided tissue regeneration" OR "soft tissue management" OR "soft tissue correction" OR "connective tissue graft" OR "autogenous graft" OR "soft tissue graft" #3 TITLE-ABS-KEY("peri-implant" OR "dental implant*" OR "implant*" OR "dental implantation") #1 AND #2 AND #3
Cochrane Library	#1 (collagen matrix):ti,ab,kw #2 (collagen matrices):ti,ab,kw #3 (collagen-based matrix):ti,ab,kw #4 (mucograft):ti,ab,kw #5 (mucoderm):ti,ab,kw #6 (xenogeneic graft material):ti,ab,kw #7 (acellular dermal matrix):ti,ab,kw #8 (extracellular matrix):ti,ab,kw #9 (extracellular membrane):ti,ab,kw #10 (#1 OR #2 OR #3 OR #4 OR #5 OR #6 OR #7 OR #8 OR #9) #11 (soft tissue augmentation):ti,ab,kw #12(soft tissue grafting):ti,ab,kw #13 (soft tissue volume):ti,ab,kw #14 (soft tissue thickness):ti,ab,kw #15 (soft tissue thickening):ti,ab,kw #16 (guided tissue regeneration):ti,ab,kw #17 (soft tissue management):ti,ab,kw #18(autogenous graft):ti,ab,kw #19 (connective tissue graft):ti,ab,kw #20 (soft tissue graft):ti,ab,kw #21(soft tissue correction):ti,ab,kw #22 (#11 OR #12 OR #13 OR #14 OR #15 OR #16 OR #17 OR #18 OR #19 OR #20 OR #21) #10 AND #22
LILACS	#1 (collagen matrix) #2 (soft tissue augmentation) #3 (dental implants) #1 AND #2 AND #3
EASY	"collagen matrix" OR "collagen matrices" OR "collagen-based matrix" OR "mucograft" OR "mucoderm" OR "fibrogide" OR "xenogeneic graft material" OR "acellular dermal matrix" OR "extracellular membrane" OR "extracellular matrix" OR "soft tissue augmentation" OR "soft tissue graft*" OR "soft tissue volume" OR "soft tissue thickness" OR "soft tissue thickening" OR "guided tissue regeneration" OR "soft tissue management" OR "soft tissue correction" OR "connective tissue graft" OR "autogenous graft" OR "dental implant"
elibrary	#1 "кoллaгeнoвый мaтpикc" or "кoллaгeнoвaя мaтpицa" or "мyкoгpaфт" or "мyкoдepм" or "фибpoмaтpикc" or "кceнoгeннaя мaтpицa" or "дepмaльный мaтpикc" or "дepмaльнaя мaтpицa" or "aцeллюляpн* мaтpи*" or "aцeллюляpн* кoллaгeнoв* мaтpи*" or "экcтpaцeллюляpн* мaтpи*" or "collagen matrix" or "collagen matrices" or "collagen-based matrix" or "mucograft" or "mucoderm" or "fibromatrix" or "xenogeneic graft material" or "acellular dermal matrix" or "extracellular membrane" or "extracellular matrix" Re-search inside #1 #2 "имплaнт*" or "имплaнтaция" or "плacтикa мягкиx ткaнeй" or "плacтикa дecны" or "пepecaдкa дecны" or "плacтикa cлизиcтoй" or "yвeличeниe дecны" or "yвeличeниe oбъeмa" or "yвeличeниe тoлщины" or "нaпpaвлeннaя peгeнepaция" or "мeнeджмeнт мягкиx ткaнeй" or "кoppeкция мягкиx ткaнeй" or "coeдинитeльнoткaнный тpaнcплaнтaт" or "ayтoгeнный тpaнcплaнтaт" or "мягкoткaнный тpaнcплaнтaт" or "dental implant*" or "soft tissue augmentation" or "soft tissue grafting" or "soft tissue volume" or "soft tissue thickness" or "soft tissue thickening" or "guided tissue regeneration" or "soft tissue management" or "soft tissue correction" or "connective tissue graft" or "autogenous graft" or "soft tissue graft"

After duplicates removal, two investigators (IA and ST) independently screened the titles and content of the abstracts (if available) to choose potentially suitable studies for set inclusion criteria. At the second stage, full-text versions of studies that met the inclusion criteria and for which the decision to be included in the review could not be made based on the title or abstract, were assessed in detail against the eligibility criteria. If some studies required more information or full-text versions were not available, the authors were contacted by the investigators. Disagreements were solved by discussion and involving a third reviewer, whose decision was determinative. All articles that did not meet the criteria were excluded with an indication of the reason.

### Data extraction

Information was extracted from each included study on the following parameters:

- Study characteristics and conclusions (author(s), year of publication, number of centers, study design, groups, time of augmentation, immediate healing abutment placement (yes/no) and its characteristics, treatment sites, number of participants and treated sites (baseline/follow-up), age of participants, smokers acceptance, follow-up period, outcomes and summary results) (Table 2).
- Intervention characteristics (surgical technique, time of augmentation, recipient site formation, type of XCM, size/volume/thickness of XCM, donor site, harvesting technique, size/volume/thickness of CTG) (Table 3).
- Soft tissue thickness outcome (time of augmentation, follow-up period, measurement technique, outcomes of soft tissue thickness/volume, changes in soft tissue thickness/volume) (Table 4).
- Secondary outcomes (KMW, surgery time, PROMs, aesthetic outcomes, peri-implant tissue health, complications) (Tables 5 and 6).

**Table 2:** Study characteristics and conclusions (RCT: randomized clinical trial, XCM: xenogeneic collagen matrix, CTG: connective tissue graft, PROMs: Patient-reported outcomes measures, KT: keratinized tissue, VCMX: volume-stable collagen matrix)

	**Type of Study**	**Groups**	**Time of augmentation**	**Immediate healing abutment placement (yes/no) and its characteristics**	**Site of treatments**	**Participants/Sites (Enrolment)**	**Participants/Sites (Analysis)**	**Smokers Accepted**	**Age of participants (years)**	**Follow-up (months)**	**Outcomes**	**Summary results (study conclusion)**
Cairo et al. 2017 [25]	Single-center RCT	XCM vs CTG	Second stage	Yes (no information)	XCM 17 – upper jaw 13 – lower jaw CTG 27 – upper jaw 3 – lower jaw	60/60	58/58 2 patients with 2 implants drop out (XCM group)	Yes (< 10 cigarettes per day)	XCM 50.3 ± 12.4 CTG 48.3 ± 11.8	3, 6 months	1) Width of keratinized mucosa 2) Buccal mucosa thickness 3) Surgery time 4) PROMs 5) Periodontal health	CTG was more effective than XCM for improving horizontal soft tissue thickness at implant site XCM is associated with shorter surgical time, lower post-operative morbidity, less anti-inflammatory tablets consumption and higher final patient satisfaction than CTG
Cosyn et. al. 2021 [26]	Multi-center RCT	XCM vs CTG	During implant surgery	Yes 40 patients (CAD/CAM provisional acrylic restoration) XCM 7 patients with healing abutments 2 patient acrylic crown 24 h after the surgery CTG 8 patients with healing abutments 3 patient acrylic crown 24 h after the surgery	Premaxilla (15–25) XCM 9 – central incisors 7 – lateral incisors 2 – canines 12 – premolars CTG 8 – central incisors 8 – lateral incisors 3 – canines 11 – premolars	60/60	59/59 1 implant in CTG was lost at 1-week follow-up because of mobility	No	XCM 48.2 ± 16.3 CTG 50.1 ± 17	3 months	1) Buccal soft tissue profile 2) Surgery time 3) Graft dimensions, wound closure 4) PROMs 5) Periodontal health 6) Aesthetic outcomes	Sites treated with XCM demonstrated more shrinkage during the early healing phase than CTG XCM resulted in more marginal bone loss, deeper pockets, and more mid-facial recession than CTG
Puzio et al. 2017 [27]	Single-center RCT	No graft (I) vs XCM (before (IIa) and after (IIIa) implant placement) vs CTG (before (IIb) and after (IIIb) implant placement)	3 months before or 3 months after implant placement without uncovering	No	No graft 15– upper jaw XCM 24 – upper jaw 4 – lower jaw CTG 29 – upper jaw 1 – lower jaw	No graft I: 15/15 XCM IIa: 13/15 IIIa: 12/15 CTG IIb: 14/15 IIIb: 14/15	No graft I: 15/15 XCM IIa: 13/15 IIIa: 12/15 CTG IIb: 14/15 IIIb: 14/15	Yes (10 cigarettes per day)	No graft I: 43.3 ± 17.4 XCM IIa: 43.7 ± 13.7 IIIa: 42.1 ± 15.3 CTG IIb: 38.1 ± 16.5 IIIb: 41.1 ± 11.9	3, 12 months	1) Soft tissue thickness 2) Gingival biotype	XCM offers an alternative method to connective tissue grafts in gingival augmentation procedures, but with a lower value of soft tissue increase
Thoma et al. 2016 [15]	Single-center RCT	XCM vs CTG	Second stage without uncovering	No	XCM 10 – upper jaw CTG 9 – upper jaw 1 – lower jaw	20/20	20/20	Yes (< 10 cigarettes per day)	XCM 43.8 ± 13.2 CTG 42.7 ± 19.1	1,3 months	1) Soft tissue thickness 2) Width of keratinized tissue 3) Surgery time 4) PROMs 5) Periodontal parameters 6) Safety evaluations 7) Histology and histomorphometry	The use of volume-stable collagen matrix and CTG for peri-implant soft tissue augmentation rendered a similar result in both groups PROMs did not reveal relevant differences between the two groups
Zeltner et al. 2017 [28]	Single-center RCT	XCM vs CTG	Second stage without uncovering	No	XCM 10 – upper jaw CTG 9– upper jaw 1 – lower jaw	20/20	20/20	Yes (< 10 cigarettes per day)	XCM 43.8 ± 13.2 CTG 42.7 ± 19.1	1,3 months	Volumetric changes of soft tissues	The use of the volume-stable collagen matrix for soft tissue augmentation at implant sites resulted in a non-inferior increase of soft tissue volume compared to the use of an autogenous subepithelial connective tissue graft
Baldi et al. 2020 [29]	Multicenter RCT	No graft vs XCM vs CTG	Second stage	Yes (healing abutment)	No graft 3—upper jaw 9 – lower jaw XCM 9 – upper jaw 3 – lower jaw CTG 6 – upper jaw 6 – lower jaw	36/36	33/31 Two implants failed (CTG group)	Accepted	No graft 53.9 ± 7.8 XCM 51.1 ± 8.2 CTG 47.5 ± 5.2	1.5, 6 months	1) Width of the keratinized mucosa 2) Facial soft tissue level 3) PROMs 4) Marginal bone level	Using CTG can attain significant gains in facial soft tissue height and keratinized mucosa width as compared to no-graft controls at six months
Ashurko et.al. 2022 [30]	Single-center RCT	XCM vs CTG	During implant surgery	No	Distal part of the lower or upper jaw	30/30	30/30	Yes (< 10 cigarettes per day)	XCM 38.67 ± 4.88 CTG 38.67 ± 6.99	3 months	1) Soft tissue thickness gain 2) Width of keratinized mucosa 3) Severity of pain syndrome 4) Severity of the collateral edema 5) Quality of life 6) Histology and histomorphometry	Using of CTG provides statistically significant superior soft tissue thickness gain than XCM for soft tissue augmentation procedures around implants
Hélio et al. 2019 [31]	Single-center RCT	XCM vs CTG	During implant surgery	No	Distal part of lower jaw	12/24	12/24	Yes (< 10 cigarettes per day)	46 (28–68)	2–3 months	1) Soft tissue thickness 2) Width of keratinized tissue 3) Histomorphometry	Both grafts resulted in increased the width and thickness of KM

**Table 3:** Intervention characteristics

	**Surgical technique**	**Time of augmentation**	**Recipient site formation**	**Graft characteristics**				
				**XCM**		**CTG**		
				**Type**	**Size/Volume/Thickness**	**Donor site**	**Harvesting technique**	**Size/Volume/Thickness**
Cairo et al. 2017 [25]	Incision: crestal or slightly lingual horizontal incision XCM: double layer of XCM. Both layers were sutured at supra-periosteal buccal tissue with resorbable sutures CTG: A standard 1-mm thick CTG was sutured at buccal supra-periosteal tissue The flap was sutured to completely cover XCM or CTG	Second stage	Split-thickness	Mucograft® (Geistlich Pharma AG, Wolhusen, Switzerland)	Thickness: 6 mm	Palate/tuber	23 cases—trap door approach 4 cases – single incision technique 2 cases—de-epithelized free gingival graft 1 case – graft from maxillary tuberosity	Thickness: 1 mm
Cosyn et. al. 2021 [26]	Incision: crestal incision at the single tooth gap and sulcular incisions at the neighboring teeth CTG: CTG was harvested from the palatal mucosa XCM: The thickness of the graft was adapted to the defect with a scalpel. Following a superficial incision to release muscle tension, the graft was brought into the buccal envelope and fixed with two single sutures onto the buccal mucosa	During implant surgery	Full-thickness	Fibro-Gide® (Geistlich Pharma AG, Wolhusen, Switzerland)	Width: 9.67 mm (8.29–11.04) Length: 6.73 mm (5.96–7.51) Thickness: 3.25 mm (2.83–3.67)	Palate	Single incision technique	Width: 9.62 (8.24–11.00) Length: 6.62 (5.84–7.39) Thickness: 2.40 (1.98–2.82)
Puzio et al. 2017 [27]	Incision: “envelope technique” without making vertical incisions XCM: Collagen matrix was cut into a shape and size that corresponded to the surgical site and was stabilized with mono-filament resorbable sutures. The mucosal flap was moved to completely cover the matrix CTG: CTG was taken from the hard palate in the area between the first premolar and the first molar. It was sutured to the recipient bed with periostitic sutures. The graft was covered with a flap, which was coronally repositioned	3 months before (IIa/IIb) or 3 months after (IIIa/IIIb) implant placement a = XCM b = CTG	Split-thickness	Mucograft® (Geistlich Pharma AG, Wolhusen, Switzerland)	Not reported	Palate	Single incision technique	Not reported
Thoma et al. 2016 [15]	Incision: a flap was prepared between the ridge crest and the buccal aspect without elevated of periosteum XCM: the matrix was shaped to match the desired size in the recipient bed CTG: an autogenous connective tissue graft was harvested from the palate The XCM/CTG was positioned under the elevated buccal flap and secured with sutures	Second stage without uncovering	Split-thickness	Fibro-Gide® (Geistlich Pharma AG, Wolhusen, Switzerland)	Thickness: 8 mm	Palate	Single incision technique	Not reported
Zeltner et al 2017 [28]	Incision: flap was elevated on top of the ridge and on the lingual side XCM: the matrix was shaped to match the desired size in the recipient bed CTG: an autogenous connective tissue graft was harvested according to standard techniques XCM/CTG was positioned in the pouch and immobilized with a horizontal mattress suture to the lingual flap. Primary wound closure was achieved with horizontal mattress and single interrupted sutures	Second stage without uncovering	Split-thickness	Fibro-Gide® (Geistlich Pharma AG, Wolhusen, Switzerland)	Thickness: 8 mm	Palate	Single incision technique	Not reported
Baldi et al. 2020 [29]	Incision: the recipient site was prepared with a crestal incision and with creating a flap on the buccal side XCM: matrix shaped to adapt to the implant site CTG: a size of graft modified according to the receiving site, harvested from the palate; No graft: the soft tissues were displaced buccally and stabilized with the healing abutment but no graft The grafts were stabilized to the periosteum with a horizontal suture. Soft tissues were sutured to the lingual/palatal flap with 5–0/4–0 resorbable sutures	Second stage	Split-thickness	Derma® (Osteobiol, Tecnoss, Coazze, Torino, Italy)	Thickness: 2 mm	Palate	Not reported	Thickness: 2 mm
Ashurko et.al. 2022 [30]	Incision: crestal incision with a periostotomy was performed from the buccal side CTG: graft was harvested from tuber area of the upper jaw using a double incision technique XCM: the collagen matrix was modeled according to the shape of the recipient bed The XCM/SCTG was positioned with overlapping the alveolar ridge under flaps from the occlusal and buccal sides. Fixation was performed to the buccal and oral flap using mattress (horizontal) suture	During implant surgery	Full-thickness	FibroMATRIX ® (LLC “Cardioplant”, Russia)	Thickness: 1.5 mm Volume: 572.3 ± 166 mm^3^	Tuber	Double incision technique	Thickness: 1.5 mm
Hélio et al. 2019 [31]	Incision: standard crestal incision CTG: a mucotome with 8 mm in diameter and marking 4 mm of depth was used for graft harvesting XCM: the size of matrix was the same with CTG CTG/XCM were fixed by two simple sutures with mononylon 5.0, one to vestibular flap and the other to lingual flap. Flaps were closed primarily with simple sutures completely covering the grafts	During implant surgery	Full-thickness	Mucograft Seal ® (Geistlich Pharma AG, Wolhusen, Switzerland)	Thickness: 4 mm	Palate	Mucotome	Thickness: 4 mm

**Table 4:** ‘Summary of findings’

Certainty assessment							№ of patients		Effect		Certainty	Importance
**№ of studies**	**Study design**	**Risk of bias**	**Inconsistency**	**Indirectness**	**Imprecision**	**Other considerations**	**xenogeneic collagen matrix**	**autogenous subepithelial connective tissue graft**	**Relative** **(95% CI)**	**Absolute** **(95% CI)**		
**Soft tissue thickness gain (follow-up: median 3 months)**												
7	randomised trials	serious^a^	not serious	not serious	not serious	none	148	149	-	MD **0.032 lower** (0.47 lower to 0.17 lower)	⨁⨁⨁◯ Moderate	IMPORTANT
**Soft tissue thickness gain (subgroup buccal) (follow-up: median 3 months)**												
6	randomised trials	not serious	not serious	not serious	not serious	none	116	118	-	MD **0.32 lower** (0.52 lower to 0.13 lower)	⨁⨁⨁⨁ High	IMPORTANT
**Soft tissue thickness gain (subgroup crestal) (follow-up: median 3 months)**												
3	randomised trials	serious^a^	not serious	not serious	not serious	none	32	31	-	MD **0.3 lower** (0.61 lower to 0)	⨁⨁⨁◯ Moderate	IMPORTANT
**Soft tissue thickness gain (subgroup digital) (follow-up: median 3 months)**												
5	randomised trials	not serious	not serious	not serious	not serious	none	89	90	-	MD **0.3 lower** (0.5 lower to 0.11 lower)	⨁⨁⨁⨁ High	IMPORTANT
**Soft tissue thickness gain (subgroup analogue) (follow-up: median 3 months)**												
3	randomised trials	serious^a^	not serious	not serious	not serious	none	59	59	-	MD **0.35 lower** (0.6 lower to 0.09 lower)	⨁⨁⨁◯ Moderate	IMPORTANT

*CI* confidence interval, *MD* mean difference

Explanations

^a^The quality of evidence had to be reduced because the study by Hélio et al. 2019 was classified as a high risk of bias

**Table 5:** Soft tissue thickness outcome

	**Time of augmentation**	**Follow-up (months)**	**Measurement technique**	**Outcome soft tissue thickness/volume (mm/mm**^**3**^**)**							**Changes in soft tissue thickness/volume (mm/mm**^**3**^**)**						
Cairo et al. 2017 [25]	Second stage	3,6	Analogue technique 1,0 mm coronal to the MGJ using an injection needle with a silicon stop and digital caliper with 0.01mm of accuracy			XCM (mm)		CTG (mm)								XCM (mm)	CTG (mm)
				Baseline		2.1 ± 0.63		2.1 ± 0.59			3 months					-	-
				3 months		2.8 ± 0.7		3.1 ± 0.5			6 months					0.9 ± 0.2	1.2 ± 0.3
				6 months		3.0 ± 0.7		3.4 ± 0.6									
Cosyn et. al. 2021 [26]	During implant surgery	3	Digital technique (intraoral scanner) Study-relevant area from 0.5 mm below the soft tissue margin to 4 mm more apical. In the mesio-distal dimension, the area reached from the mesial to the distal line angle of the implant crown			XCM (mm^3^)		CTG (mm^3^)								XCM (mm, 95% CI)	CTG (mm, 95% CI)
				Baseline		28.07		28.63			Post-surgery					1.90 (1.63–2.18)	1.43 mm (1.15–1.70)
				Post-surgery		50.93		39.85			3 months					0.85 (0.58–1.13)	1.15 (0.88–1.43)
				3 months		22.55		30.86			Graft shrinkage					1.05 (0.79–1.31)	0.27 (0.01–0.53)
Puzio et al. 2017 [27]	3 months before (IIa/IIb) or 3 months after (IIIa/IIIb) implant placement a = XCM b = CTG	3,12	Ultrasonic device (Pirop®, Echoson) 1) Point 1: on the line, connecting the cemento-enamel junctions of both adjacent teeth on the gingival margin 2) Point 2: on the mucogingival junction along the axis of the future implant			I (mm)	IIa (mm)	IIb (mm)	IIIa (mm)	IIIb (mm)			I (mm)	IIa (mm)	IIb (mm)	IIIa (mm)	IIIb
				Baseline	Point 1	1.39 ± 0.65	1.30 ± 0.46	1.30 ± 0.23	1.21 ± 0.49	1.15 ± 0.40	3 months	Point 1	0.23 ± 0.6	0.44 ± 0.6	0.89 ± 0.6	0.62 ± 0.9	0.95 ± 0.7
					Point 2	1.10 ± 0.44	1.04 ± 0.47	0.75 ± 0.26	1.01 ± 0.41	0.90 ± 0.30		Point 2	0.21 ± 0.4	0.38 ± 0.5	0.64 ± 0.5	0.48 ± 0.4	1.01 ± 0.7
				3 months	Point 1	1.62 ± 0.50	1.74 ± 0.37	2.19 ± 0.55	1.83 ± 0.83	2.10 ± 0.77	12 months	Point 1	0.7 ± 0.8	1.16 ± 0.7	1.76 ± 0.7	0.89 ± 0.6	1.52 ± 1.0
					Point 2	1.31 ± 0.41	1.43 ± 0.56	1.39 ± 0.48	1.48 ± 0.44	1.91 ± 0.78		Point 2	0.35 ± 0.6	1.0 ± 0.7	1.36 ± 0.6	0.57 ± 0.6	1.15 ± 0.5
				12 months	Point 1	2.10 ± 0.66	2.46 ± 0.75	3.06 ± 0.61	2.10 ± 0.50	2.68 ± 0.96							
					Point 2	1.46 ± 0.34	2.04 ± 0.61	2.11 ± 0.70	1.57 ± 0.52	2.05 ± 0.56							
Thoma et al. 2016 [15]	Second stage without uncovering	1,3	Analogue technique An individualized stent was fabricated by CAD/CAM technology with three standardized openings (occlusal, buccal, apical). Endodontic instrument introducing in the openings in two areas: penetrating the mucosa to the bone and on top of mucosa					XCM (mm)		CTG (mm)				XCM (mm)		CTG (mm)	
				Baseline		Occlusal		3.4 ± 1.0		4.2 ± 1.9	1 month		Occlusal	0.9 ± 1.4		0.9 ± 1.5	
													Buccal	1.7 ± 1.6		1.6 ± 2.3	
													Apical	2.5 ± 1.6		1.5 ± 2.6	
						Buccal		2.9 ± 1.5		4.1 ± 2.0	3 months		Occlusal	1.4 ± 1.4		0.8 ± 1.8	
						Apical		2.6 ± 2.3		3.4 ± 1.8			Buccal	1.1 ± 1.4		0.8 ± 2.2	
													Apical	0.9 ± 1.9		1.6 ± 2.6	
											1 to 3 months		Occlusal	0.5 ± 1.9		0.1 ± 1.4	
													Buccal	0.4 ± 1.4		0.4 ± 2.0	
													Apical	1.7 ± 1.7		0.6 ± 2.0	
Zeltner et al 2017 [28]	Second stage without uncovering	1,3	Digital-assisted technique with analogue impressions Two specific regions (ROI) of interest: Crestal: determined by the midcrestal line and the gingival margins of the adjacent teeth Buccal: characterized by a trapezoid shape and defined as the area between the gingival margins of the adjacent teeth, the mucogingival junction as apical and the interproximal areas as lateral borders					XCM (mm^2^, Q1; Q3)		CTG (mm^2^, Q1; Q3)				XCM (mm)		CTG (mm)	
				Baseline		Crestal		24.8 (23.8; 26.9)		23.7 (21.2; 26.2)	1 month		Crestal	0.56 ± 0.41		0.66 ± 0.68	
													Buccal	1.16 ± 0.72		1.05 ± 0.61	
											3 months		Crestal	0.27 ± 0.26		0.42 ± 0.74	
						Buccal		32.2 (31.6; 33.1)		29.2 (24.6; 31.4)			Buccal	0.77 ± 0.74		0.79 ± 0.45	
											1 to 3 months		Crestal	-0.29 ± 0.24		-0.24 ± 0.33	
													Buccal	-0.39 ± 0.22		-0.25 ± 0.26	
Baldi et al. 2020 [29]	Second stage	1.5, 6 months	Analogue technique Facial soft tissue level (FST): the distance between the mid-facial soft tissue level and a reference line connecting the facial soft tissue level of the adjacent teeth	Not reported									XCM (mm)	CTG (mm)		No graft (mm)	
											1.5 months		0.34 ± 0.13 (SE)				
											(95% Cl 0.05; 0.63)		0.50 + 0.22 (SE)				
											(95% Cl -0.07; 1.07)		-0.40 ± 0.23 (SE)				
											(95% Cl -0.91; -0.09)						
											6 months		0.32 ± 0.21 (SE)				
											(95% Cl -0.13; 0.78)		0.35 ± 0.30 (SE)				
											(95% Cl -0.44; 1.34)		-0.95 ± 0.42 (SE)				
											(95% Cl -1.8; -0.02)						
Ashurko et.al. 2022 [30]	During implant surgery	3	Digital technique with analogue impressions Plaster models were scanned with the laboratory scanner. With GOM Inspect the 3D-models compared and the buccal contour changes evaluated at 3 equidistant points			XCM (mm)		CTG (mm)						XCM (mm)		CTG (mm)	
				Baseline		1.61 ± 0.07		1.63 ± 0.07									
											3 months			1.18 ± 0.11		1.55 ± 0.11	
				3 months		2.81 ± 0.11		3.16 ± 0.11									
Hélio et al. 2019 [31]	During implant surgery	2 to 3	Analogue technique A puncture with a short carpule needle with an endodontic rubber cursor was performed in the center of the future prosthetic crown			XCM (mm)		CTG (mm)			Not reported						
				Baseline		2.12 ± 0.33		2.06 ± 0.33									
				3 months		2.61 ± 0.43		2.98 ± 0.5

**Table 6:** Secondary outcomes (BL: Bone level, PD: Probing depth, BoP: Bleeding on probing, CAL: clinical attachment level, Pl: Plaque, PES: pink aesthetic score, MSI: mucosal scarring index)

	**Width of Keratinized mucosa (mm)**						**Surgery time (min)**	**Patient-reported outcomes measures (PROMs)**	**Aesthetic outcomes (pink aesthetic score/ white aesthetic score)**	**Periimplant tissue health**			**Complications**
Cairo et al. 2017 [25]			XCM				XCM 35.5 ± 9.4 CTG 51.7 ± 7.0	VAS value was 17 ± 13 (XCM) and 35 ± 23 (CTG) After 7 days, patients in the XCM group experienced also significantly lower intensity of post-surgical pain than the CTG group (13.0 ± 10 vs 37.0 ± 15) and lower number of unconformable days (1.2 ± 0.7 vs 2.4 ± 0.7) At 2 weeks, the only significant difference was the higher number of sites with edema (20 vs 7 sites) in the CTG group At 6 months all patients were highly satisfied, with 95 ± 5 mean VAS value in XCM group and 91 ± 9 in the CTG group	At 6 months all patients were satisfied in term of aesthetic outcomes with no difference between groups	BL, PD, BoP—no statistically significant difference			1 case of 1 mm soft tissue recession 6 months post.op
	Baseline		3.1 ± 1.2			3.5 ± 1.1							
	3 months		3.7 ± 1.1			4.0 ± 1.7							
	6 months		4.3 ± 1.2			4.4 ± 1.5							
	∆ 6 months		1.1 ± 0.8			0.9 ± 0.8							
Cosyn et. al. 2021 [26]	Not reported						XCM 13.00 (8.51–17.49) CTG 22.03 (17.54–26.52)	There were no significant differences between the groups in terms of post-operative bleeding, pain and edema, but there was for post-operative hematoma (10.23 in the CTG group and 3.67 in the XCM group) There was no significant difference between the groups with number of analgesics (5.24 and 4.47 in the CTG and XCM group respectively)	Mean mid-facial recession was 0.16 mm in the CTG group and 0.91 mm in the XCM group at 3 months PES and MSI with no statistically significant difference between the groups		XCM (95% CI)	CTG (95% CI)	In the CTG group: 1) 1 patient experienced intolerable pain and oedema 2) 1 patient with 1 implant showed mobility after 1 week and was removed 3) 2 patients showed a wound dehiscence after 1 week In the XCM group: 1)1 patient with heavy post-operative bleeding 2) 1 patient showed a wound dehiscence after 1 week
										BL	0.72 (0.39–1.04)	0.34 (0.01–0.67)	
										PD	3.66 (3.29–4.03)	3.36 (2.99–3.73)	
										PI, BoP	no statistically significant difference		
Puzio et al. 2017 [27]	Not reported						Not reported	Not reported	Not reported	Not reported			Not reported
Thoma et al. 2016 [15]	No significant differences (data not shown)						XCM 39.0 (33.0; 51.0) CTG 34.0 (27.0; 45.0)	A higher VAS score for CTG between day 1 and day 3 post surgery was demonstrated without statistically significant difference. At suture removal, median overall scores of the OHIP-G14 questionnaire for CTG were higher than for XCM without reaching statistically significant differences. Median physical pain was 100% higher in group CTG compared to group XCM. The greatest differences between the groups were found for physical pain and social disability	Not reported	PD, BoP, CAL, PI—no statistically significant difference			In 2 patients (1 in each group) no or only limited volume gain was observed
Zeltner et al 2017 [28]	Not reported						Not reported	Not reported	Not reported	Not reported			One patient could not be included in the linear volumetric evaluation because of missing stone replicas
Baldi et al. 2020 [29]		XCM		CTG	No graft		Not reported	Patients’ mean aesthetic satisfaction this was high for all groups. There were no statistically significant differences between groups in terms of aesthetic outcomes	No significant differences in PES between groups. The only significant difference in interdental papilla index was between CTG and No graft group	BL—no statistically significant difference			None
	Baseline	2.00 ± 1.2		3.87 ± 1.1	1.84 ± 1.0								
	1.5 months	2.80 ± 1.2		4.66 ± 0.5	2.00 ± 1.0								
	6 months	3.05 ± 1.3		4.91 ± 0.8	2.08 ± 1.2								
	Changes 1.5 months	0.08 ± 0.36 (SE)		0.5 ± 0.56 (SE)	0.08 ± 0.31 (SE)								
	Changes 6 months	1.05 ± 0.35 (SE)		0.8 ± 0.67 (SE)	0.16 ± 0.45 (SE)								
Ashurko et.al. 2022 [30]	No significant difference						XCM 23.4 ± 5.54 CTG 31.8 ± 6.86	No statistically significantly different with VAS score and OHIP-G14 questionnaire between groups. Edema on the 1st day was statistically significantly greater in XCM group, by day 3rd there was no difference	Not reported	Not reported			Complicated healing was observed in 2 patients of the test group. Discrepancy of the wound edges with exposure of the collagen matrix was determined
Hélio et al. 2019 [31]		XCM			CTG		Not reported	Not reported	Not reported	Not reported			Not reported
	Baseline	3.0 ± 0.65			3.21 ± 0.71								
	3 months	3.67 ± 0.79			4.41 ± 0.55								

### Assessment of the risk of bias

Two reviewers (SB and MM) independently performed the assessment of risk of bias for included studies using the Cochrane Collaboration’s tool for assessing risk of bias in randomized controlled clinical studies [22]. Seven domains were analyzed, including sequence generation, allocation concealment, blinding of participants and personnel, blinding of the outcome assessor, incomplete outcome data, selective reporting and other bias. Risk of bias judgments were categorized as low, unclear and high risk of bias.

### Quality of evidence assessment

The confidence in the evidence of effects found in the meta-analysis was assessed using the GRADE (Grading of Recommendations Assessment, Development and Evaluation) approach. Quality was assessed as high, moderate, low or very low according to GRADE. The summary table of results was constructed using GRADEpro GDT software.

### Statistical analysis

The research results were pooled using the meta library v. 5.5–0 with R programming environment v. 4.1.2 and RStudio v. 2022.07.2 (PBC, Boston). A random effects models were used for meta-analysis. Data are presented as mean or mean differences (MD) and 95% confidence interval (CI). The I^2^ statistic was used to assess the heterogeneity of studies. Study bias was assessed using a funnel plot of MD of soft tissue thickness gain at 3 months between groups versus standard error. The significance was considered at *p* < 0.05 [32].

## Results

### Study selection

**Fig. 1:**
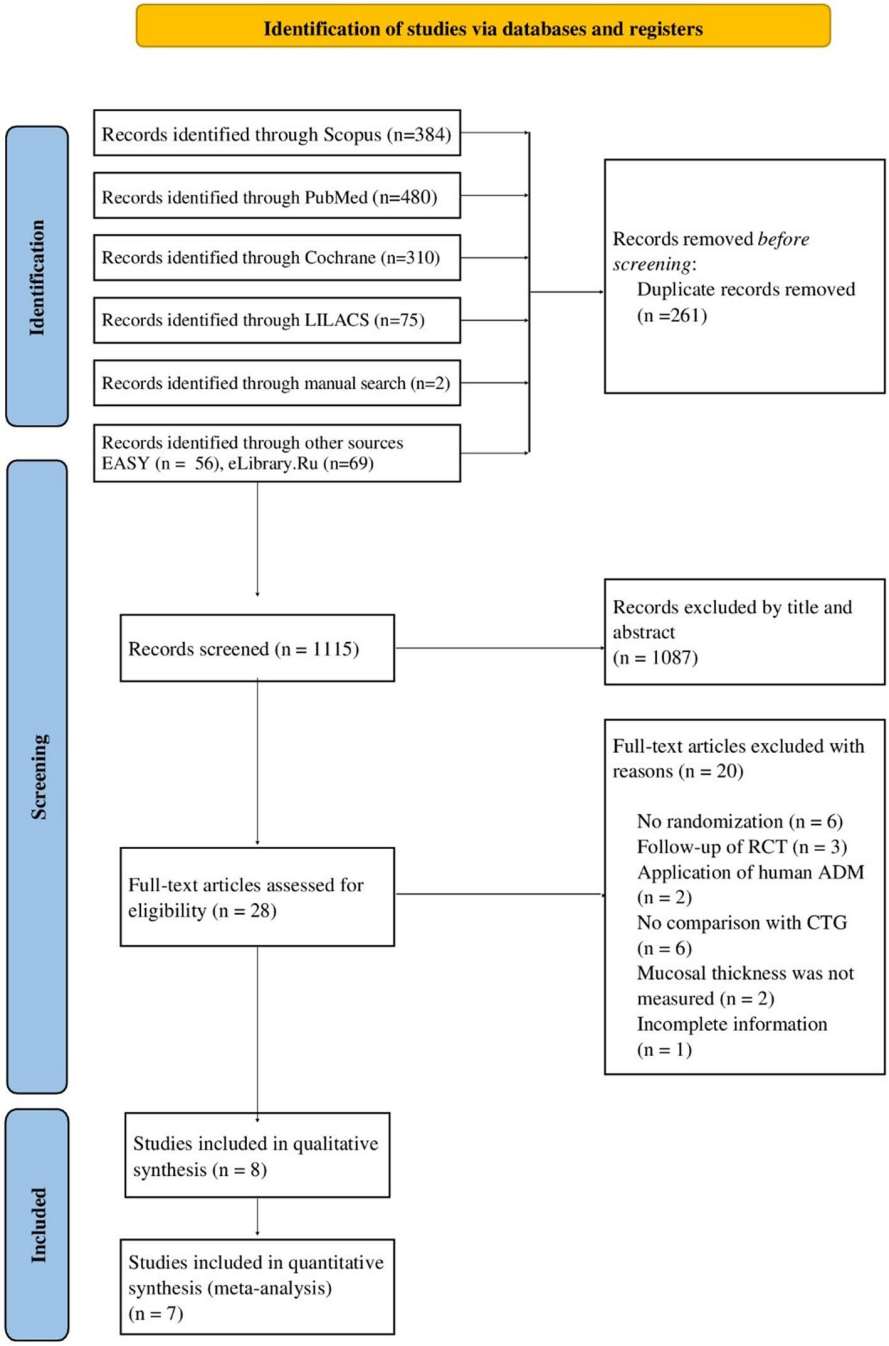
PRISMA flow diagram of study search process

The study search process is presented in Fig. 1. This review identified 1376 records in total. Following removal of duplicates, titles and abstracts of 1115 records were screened. Full-text versions of 28 articles were assessed for eligibility. After exclusion of articles that did not meet eligibility criteria, 8 studies [15, 25–31] were included in qualitative synthesis, and 7 studies [15, 25–28, 30, 31] were included in meta-analysis. Reasons for exclusion are reported in Table 7.

**Table 7:** Reasons for exclusion

	Reason for exclusion	Studies
Qualitative analysis	No randomization	De Angelis et al. [18], Schmitt et al. [16], Zafiropoulos et al. [33], Schallhorn et al. [34], Eeckhout et al. [35], Verardi et al. [36]
	Application of human ADM	Liu et al. [37], Amin et al. [38]
	No comparison with CTG	Lai et al. [39], Tavelli et al. [40], Froum et al. [41], Maiorana et al. [42], Artzi et al. [43], Zafiropoulos et al. [44]
	Gingival thickness was not measured	Santagata et al. [45], Sanz et al. [46]
	Non-interventional follow-up study	Thoma et al. [47], Huber et al. [48], Puzio et al. [49]
	Incomplete data on the timing of measurements (no measurements immediately before soft tissue augmentation)	Zuiderveld et al. [20]
Quantitative analysis	Incomplete data on measurements of gingival thickness	Baldi et al. [29]

### Study characteristics

All the included studies [15, 25–31] had RCT design. Soft tissue augmentation procedures at implant sites were described in studies involving 306 patients and 325 implants in total. All studies [15, 25–31] included at least two parallel groups comparing the use of CTG with XCM. The follow-up time ranged from 1 to 12 months. More detailed characteristics of the included studies are summarized in Tables 2 and 3.

### Qualitative analysis

**Fig. 2:**
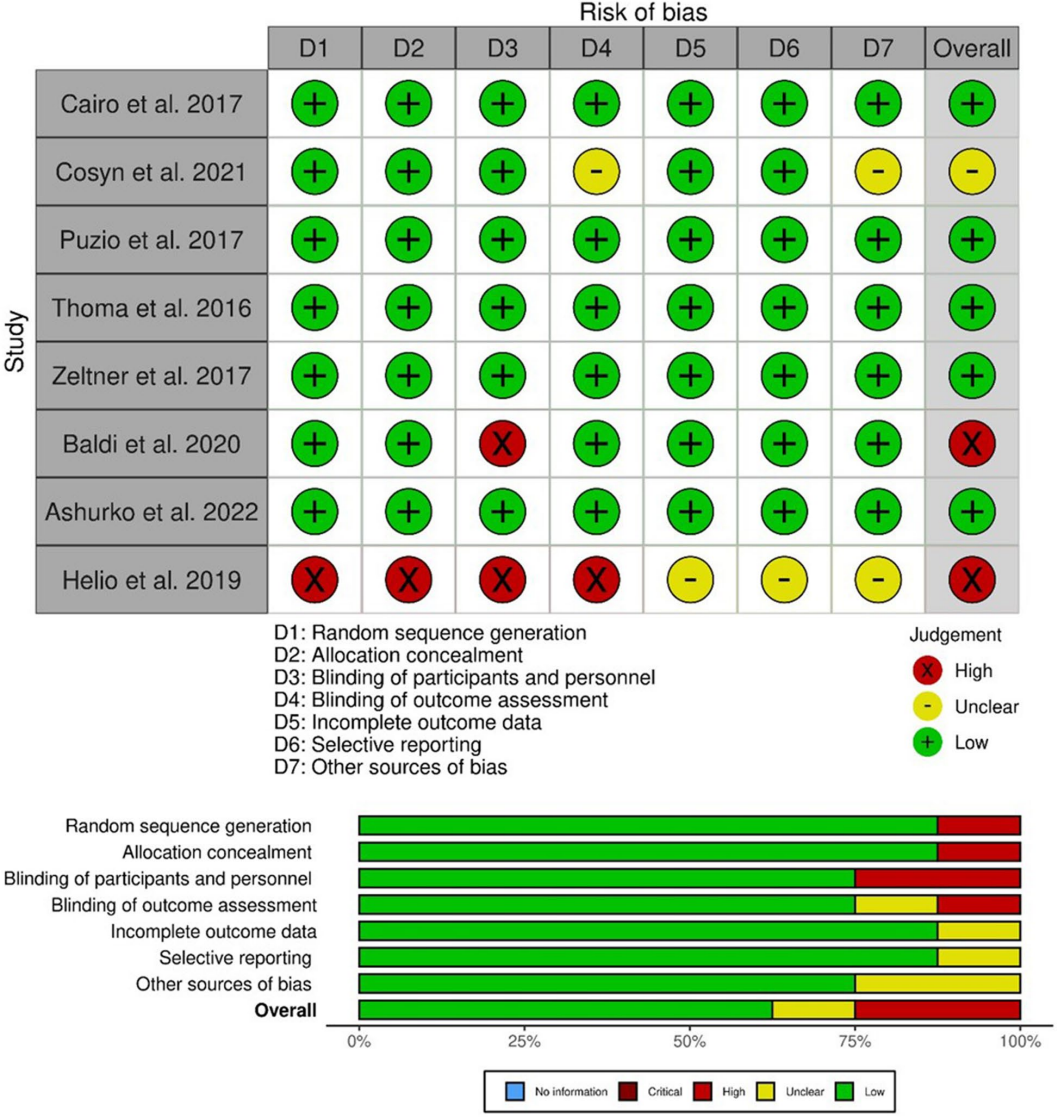
Risk of Bias assessment

The result of bias risk assessment for the included randomized clinical trials, using The Cochrane Risk of Bias Tool [22] is shown in Fig. 2. Five [15, 25, 27, 28, 30] of the included studies were considered a low risk of bias, one trial [26] was classified as a moderate risk of bias, and two studies had a high risk of bias [29, 31].

### Statistical analyses

**Fig. 3:**
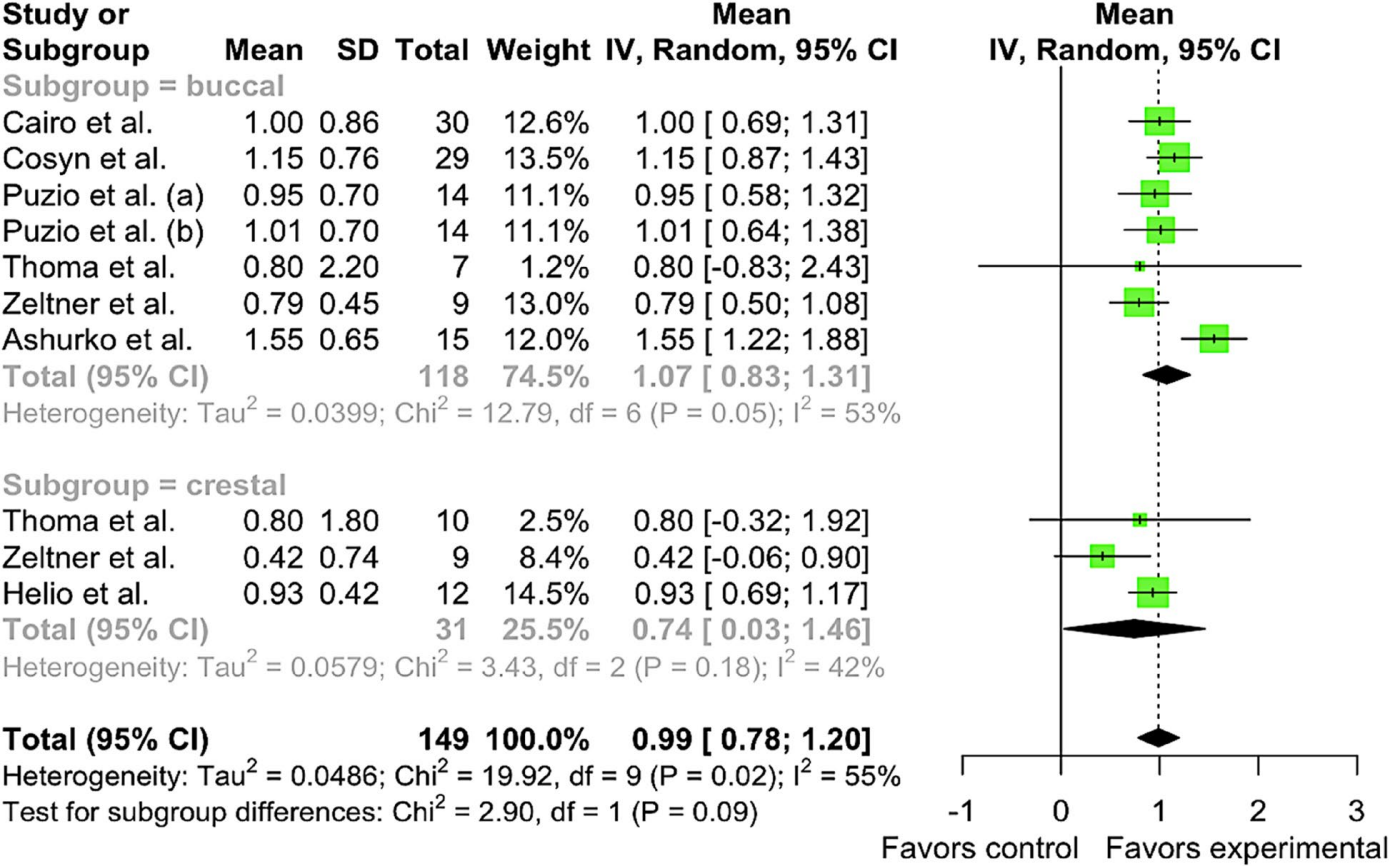
Forest plot analysis of pooled mean of 3-month gain in soft tissue thickness of CTG (control) group, random effect model meta-analysis (subgroup by position), significance at *p* < 0.05

Meta-analysis of included studies [15, 25–28, 30, 31] revealed that in the CTG group, the pooled mean 3-month soft tissue thickness gain in the buccal subgroup was μ = 1.0704 mm [95% CI: 0.8325 – 1.3084, I^2^ = 53.1%]. Heterogeneity has been found in studies. Pooled mean of CTG group 3-month soft tissue thickness gain in the crestal subgroup was μ = 0.7428 mm [95% CI: 0.0280 – 1.4577, I^2^ = 41.8%]. No differences were observed between subgroups (Chi^2^ = 2.90, df = 1, *p*-value = 0.0888). Total pooled mean of CTG was μ = 0.9881 [95% CI: 0.7803 – 1.1959] with heterogeneity I^2^ = 54.8% [95% CI: 8.0% – 77.8%, Chi^2^ = 19.92, df = 9, *p*-value = 0.0184] (Fig. 3).

**Fig. 4:**
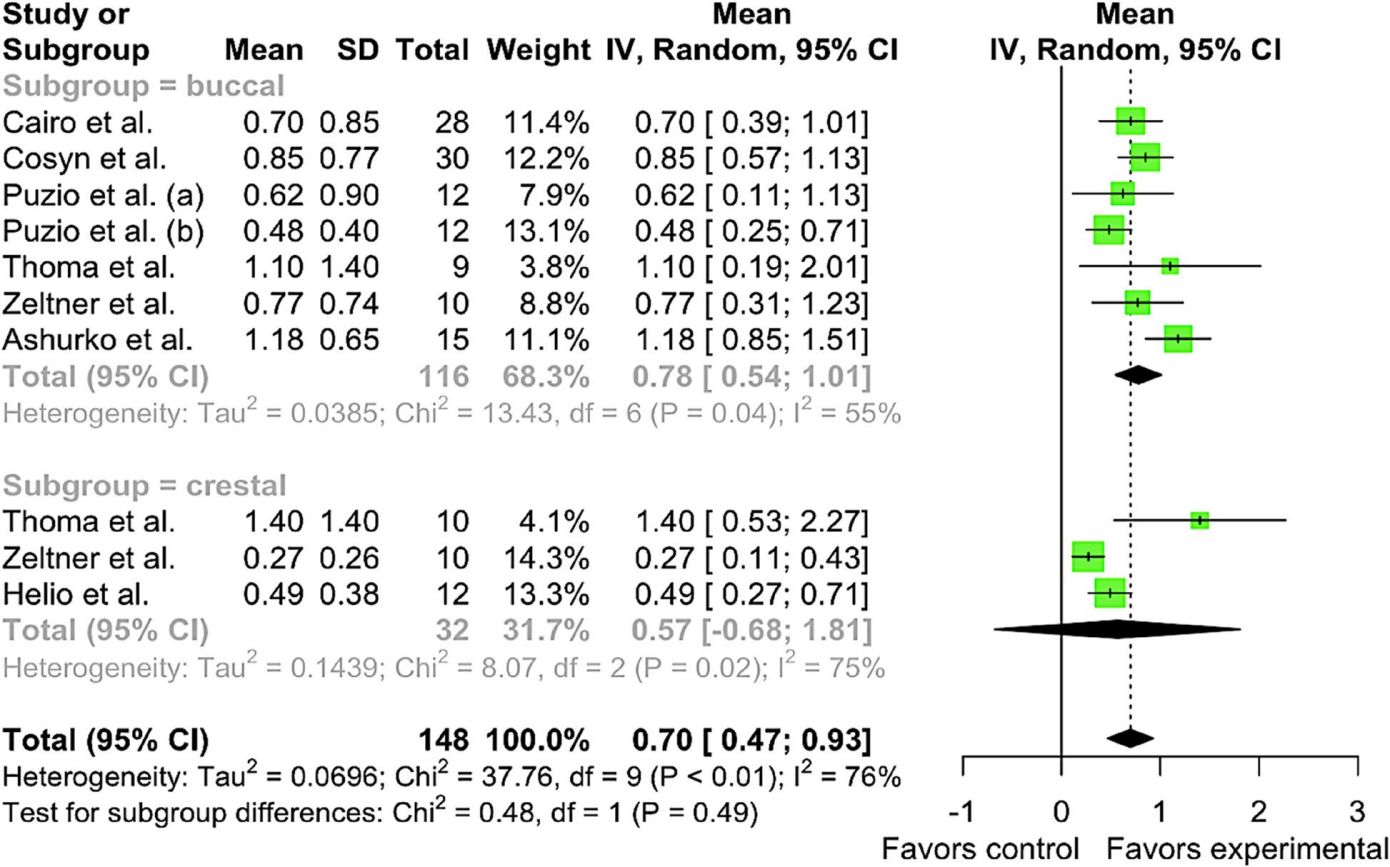
Forest plot analysis of pooled mean of 3-month gain in soft tissue thickness of XCM (experimental) group, random effect model meta-analysis (subgroup by position), significance at *p* < 0.05

Meta-analysis revealed that the pooled mean 3-month soft tissue thickness gain in buccal subgroup of XCM group was μ = 0.7778 mm [95% CI: 0.5446 – 1.0109, I^2^ = 55.3%]. Heterogeneity has been found in studies. Pooled mean of CTG group 3-month soft tissue thickness gain in the crestal subgroup was μ = 0.5661 mm [95% CI: -0.676 – 1.8084, I^2^ = 75.2%]. No differences were observed between subgroups (Chi^2^ = 0.48, df = 1, *p*-value = 0.4864). Total pooled mean of CTG was μ = 0.6972 [95% CI: 0.4659 – 0.9284] with heterogeneity I^2^ = 76.2% [95% CI: 55.9% – 87.1%, Chi^2^ = 37.76, df = 9, *p*-value < 0.0001] (Fig. 4).

**Fig. 5:**
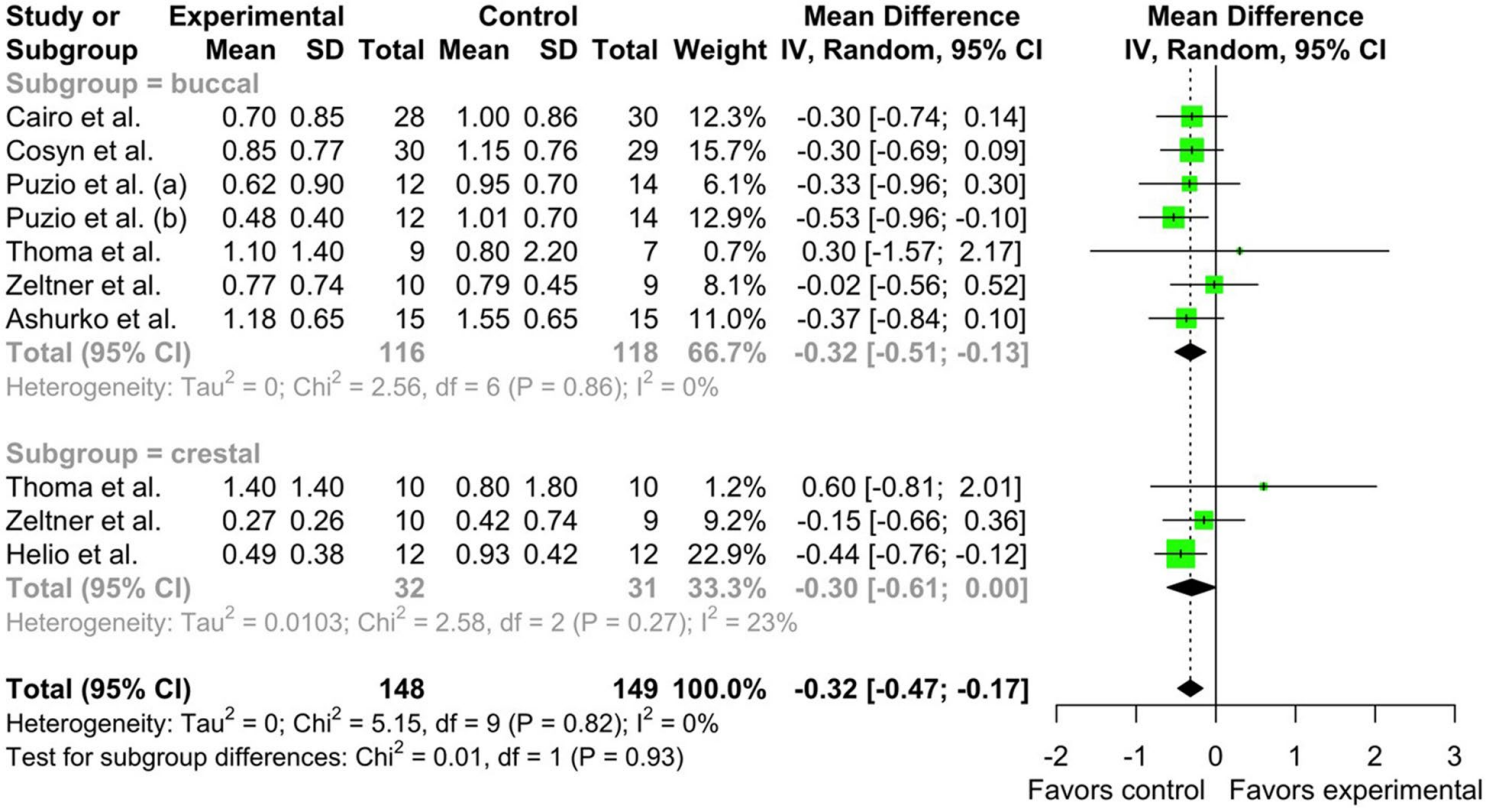
Forest plot analysis of MD of 3-month gain in soft tissue thickness of CTG (control) and XCM (experimental) groups, random effect model meta-analysis (subgroup by position), significance at *p* <0.05

When comparing groups, it has been revealed that in the buccal subgroup mean difference (MD) between XCM (experimental) and CTG (control) 3-month soft tissue thickness tissue gain was -0.3187 mm [95% CI: -0.5080 – -0.1295, I^2^ = 0%]. In crestal subgroup MD was -0.3026 mm [95% CI:—0.6074 – 0.0021, I^2^ = 22.6%.]. No differences were observed between subgroups (Chi^2^ = 0.01, df = 1, *p*-value = 0.9299) (Fig. 5).

Total MD between CTG and XCM 3-month soft tissue thickness gain was -0.3199 mm [95% CI:—0.4746 – -0.1653, z = -4.06, *p*-value < 0.0001]. Heterogeneity has not been found in studies I^2^ = 0% [95% CI: 0.0 – 62.4%, Chi^2^ = 5.15, df = 9, *p*-value = 0.8212] (Fig. 5).

**Fig. 6:**
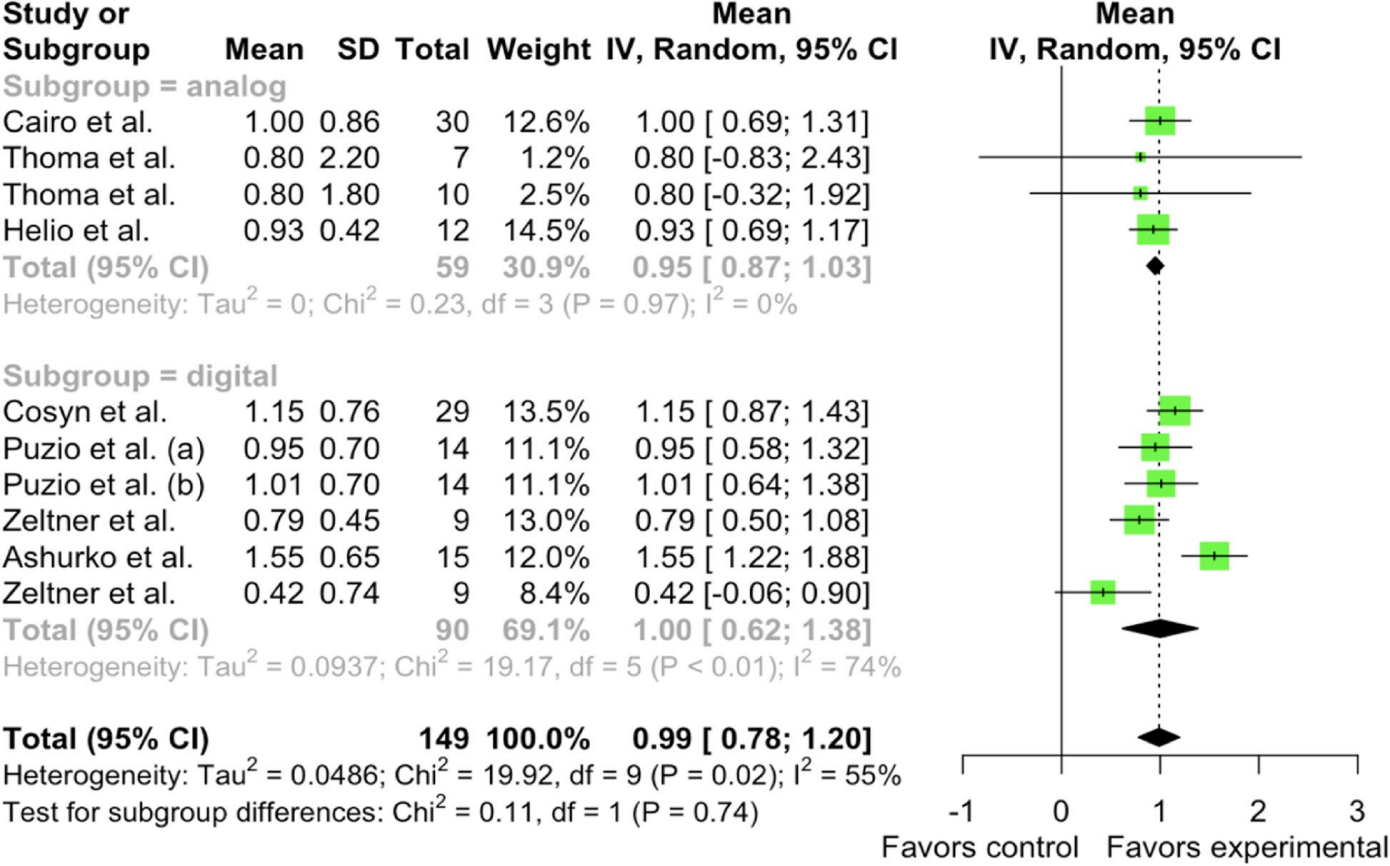
Forest plot analysis of pooled mean of 3-month gain in soft tissue thickness of CTG (control) group, random effect model meta-analysis (subgroup by methodology), significance at *p* < 0.05

In order to evaluate the impact and heterogeneity of measurement methods (analog, digital) on soft tissue thickness gain in the studies, a meta-analysis was undertaken, incorporating research studies [15, 25–28, 30, 31]. A meta-analysis revealed that within CTG group, the pooled mean 3-month soft tissue thickness gain in the analog subgroup was μ = 0.9501 mm [95% CI: 0.8668–1.0334, I^2^ = 0.0%]. In the digital subgroup of the CTG group, the pooled mean 3-month in soft tissue thickness gain was μ = 0.9992 mm [95% CI: 0.6173 – 1.3811, I^2^ = 73.9%]. Heterogeneity was identified in the studies. No significant differences were observed between the subgroups (Chi^2^ = 0.11, df = 1, *p*-value = 0.7450) (Fig. 6).

**Fig. 7:**
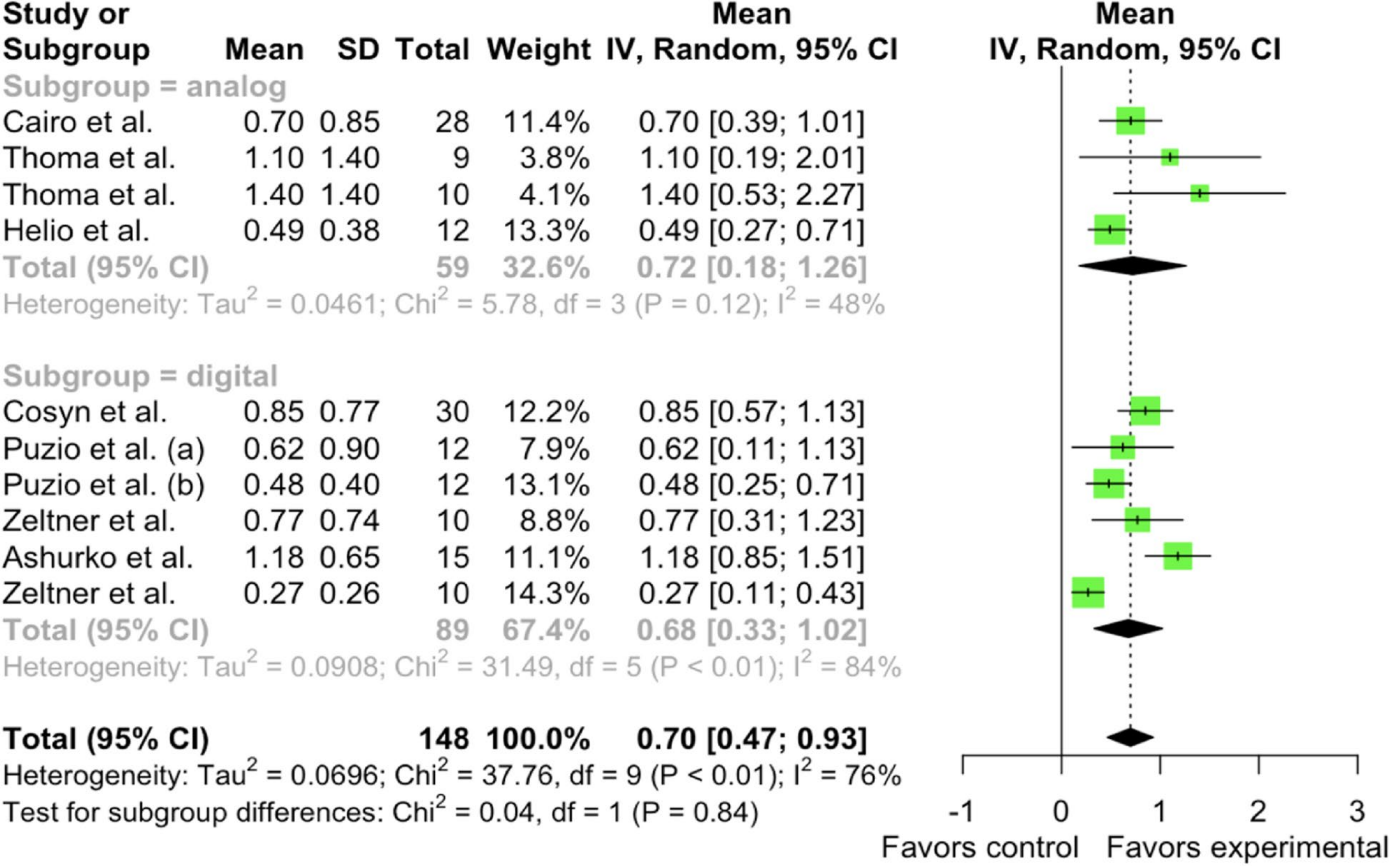
Forest plot analysis of pooled mean of 3-month gain in soft tissue thickness of XCM (experimental) group, random effect model meta-analysis (subgroup by methodology), significance at *p* < 0.05

Meta-analysis revealed that the pooled mean 3-month soft tissue thickness gain within the analog subgroup of the XCM group was μ = 0.7208 mm [95% CI: 0.1804 – 1.2611, I^2^ = 48.1%]. Heterogeneity has been found in studies. The pooled mean within the digital subgroup of the CTG group in 3-month soft tissue thickness gain was μ = 0.6771 mm [95% CI: 0.3321 – 1.0221, I^2^ = 84.1%]. No significant differences were observed between the subgroups (Chi^2^ = 0.04, df = 1, *p*-value = 0.8402) (Fig. 7).

**Fig. 8:**
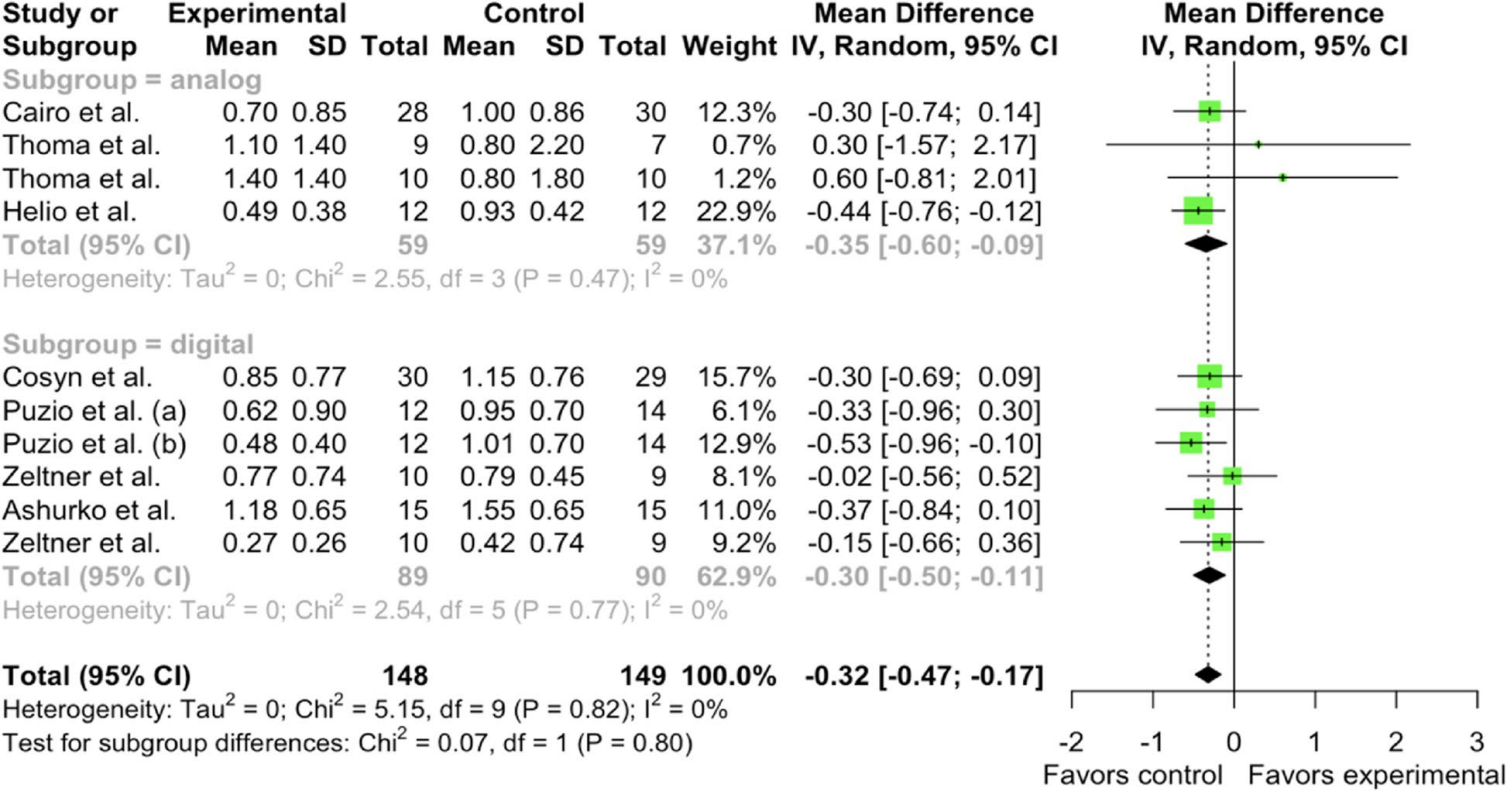
Forest plot analysis of MD of 3-month gain in soft tissue thickness of CTG (control) and XCM (experimental) groups, random effect model meta-analysis (subgroup by methodology), significance at *p* < 0.05

Furthermore, when comparing the study groups, it was revealed that in the analog subgroup mean difference (MD) between XCM (experimental) and CTG (control) 3-month soft tissue thickness tissue gain was -0.3463 mm [95% CI: -0.6001; -0.0924– -0.1295, I^2^ = 0%]. In the digital subgroup MD was -0.3044 mm [95% CI: -0.4994 – -0.1095, I^2^ = 0.0%]. No heterogeneity was found in the studies, and no significant differences were observed between the subgroups (Chi^2^ = 0.07, df = 1, *p*-value = 0.7979) (Fig. 8).

**Fig. 9:**
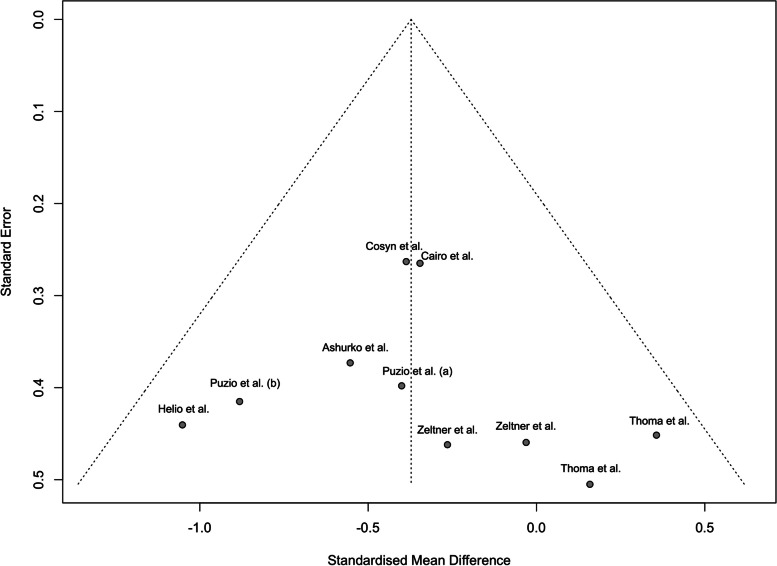
Funnel plot of SMD of 3-month gain in soft tissue thickness of CTG (control) and XCM (experimental) groups versus SE

Analysis of publication bias in studies reporting difference of 3-month soft tissue thickness gain in CTG versus XCM groups was performed by funnel plot. No significant asymmetrical distribution was found. Statistical evaluation of publication bias was not performed due to the limited number of subgroup studies (buccal n = 7, crestal n = 3) (Fig. 9).

### Quality of evidence

The quality of the evidence was assessed using the GRADE approach. The following outcomes were considered important: soft tissue thickness gain at 3 months in total and in subgroups divided by position (buccal and crestal) and by methodology (analogue and digital). As recommended, the baseline level of evidence for outcomes is high, reasons for reducing the quality of evidence are summarised in ‘Summary of findings’ table. Two authors (IA and AG) worked independently to assess the quality of evidence, disagreements were resolved by consensus. Quality of two outcomes was graded as high and quality of three outcomes was assessed as moderate according to GRADE criteria.

**Question:** Can modern XCMs provide results comparable to autogenous CTGs in increasing soft tissue thickness in the area of dental implants?

### Soft tissue thickness

#### Measurement methods of soft tissue thickness

Measurements of the mucosal thickness were performed by various methods. The analog measurement method was used by 4 authors, [15, 25, 29, 31] 3 authors used the digital method, [26, 28, 30] and in 1 study [27] ultrasound device was used.

Analog techniques for measuring soft tissue thickness differed from each other. Thus, Cairo et al. used an injection needle with a silicon stop and digital caliper with 0.01 mm of accuracy. The measurement was carried out at 1 point, which was located 1.0 mm coronal to the mucogingival junction (MGJ) [25]. Hélio et al. measured supracrestal soft tissue thickness by a puncture with a short carpule needle with an endodontic rubber cursor in the center of the future prosthetic crown [31]. Thoma et al. also measured by transmucosal probing with an endodontic instrument. There were 3 points of measurement: the occlusal, buccal and apical aspects, which were standardized by an individualized stent fabricated by CAD/CAM technology [15]. Baldi et al. conducted evaluation of facial soft tissue level (FST) measured as the distance in mm between the mid-facial soft tissue level and a reference line connecting the FST of the adjacent teeth [29].

Digital techniques for measuring soft tissue thickness were presented in three studies [26, 28, 30]. Zeltner et al. used digital-assisted technique with impressions [28]. After impressions were taken, dental stone casts were fabricated and optically scanned with a desktop 3D scanner. After importing digital models into a digital imaging software program, crestal and buccal regions of interest were identified [28]. The same method was used by Ashurko et al. who investigated buccal contour changes at 3 equidistant points (in 1-mm step) in the coronally-apical direction at the center of the alveolar ridge [30].

To analyze volumetric and profilometric changes Cosyn et al. took an optical scan by intra-oral scanner. Study-relevant areas were from 0.5 mm below the soft tissue margin to 4 mm more apical and from the mesial to the distal line angle of the implant crown [26].

Puzio et al. used an ultrasonic device (Pirop®, Echoson) [27]. The thickness of the mucosa was measured at two points: the first point was located on the line connecting the cemento-enamel junctions of adjacent teeth on the gingival margin. The second point was on the MGJ along the axis of the future implant. The volumetric changes measured in mm (in the software), which corresponded to the mean distance between the three surfaces representing the evaluated time-points [27].

### Soft tissue thickness outcomes

The baseline was defined as the time of the first measurement before any augmentation took place, except for the study by Baldi et al., [29] in which baseline mucosal thickness was not determined. In 5 studies, [15, 25, 27, 30, 31] the initial soft tissue thickness in mm was determined, except for the studies by Zeltner et al. [28] and Cosyn et al., [26] where the researchers used mucosal volume parameter in mm^3^ in a specific area as an initial measurement (Tables 5 and 6).

In the study by Cairo et al., in the XCM and CTG groups, the initial gingival thickness was comparable and amounted to 2.1 ± 0.63 and 2.1 ± 0.59, respectively [25]. After three months the mean thickness for the XCM group increased to 2.8 ± 0.7, the CTG group showed 3.1 ± 0.5. The final thickness after six months in the XCM and CTG groups was 3.0 ± 0.7 and 3.4 ± 0.6, respectively. A significant gain in soft tissue thickness was observed in both groups compared to baseline (*p* < 0.0001). Soft tissue increase was 0.9 ± 0.2 in the XCM group and 1.2 ± 0.3 mm in the CTG group, and was lower in the test group (difference -0.3 mm; 95%CI 0.5 – 0.2; *p* = 0.0001). At the same time, 79% of XCM-treated sites and 93% of CTG-treated sites achieved final soft tissue thickness ≥ 2.5 mm [25].

In the study by Puzio et al., baseline soft tissue thickness before augmentation procedure was below 2 mm in all groups [27]. Mean values of gingival thickness at point 1 were between 1.15 ± 0.40 to 1.39 ± 0.65 and at point 2 were between 0.9 ± 0.77 to 1.10 ± 0.44. There was no significant difference between all groups in case of point 1 and 2. At the 3-months follow-up at the point 1 statistically significant mucosal thickness gain was observed only between groups with no graft area (I) and CTG group 3 months after implant placement (IIIb) (0.23 mm vs. 0.95 mm; *p* = 0.042). At point 2 soft tissue thickness increased significantly in the XCM group (IIIa) and CTG group 3 months after implant placement (IIIb) (0.48 mm vs. 1.01 mm; *p* = 0.042). But there were no significant differences between XCM group (IIa) and CTG group 3 months before implant placement (IIb) (*p* = 0.654). Significant differences in thickness gain between groups I–IIIb, IIa–IIIb and IIIa–IIIb were also observed. Ultrasonic assessment after 12 months showed the highest soft tissue gain at point 1 in CTG groups: 1.76 ± 0.7 (IIb) and 1.52 ± 1.0 (IIIb). The highest increase (1.76 ± 0.7) was in the group CTG before implantation (IIb), but the difference was not significant (*p* = 0.928). The smallest increase was recorded in the group where XCM was used 3 months after implant placement (0.89 ± 0.6). But there was no significant statistical difference at point 1 after 12 months between the groups with XCM and CTG before (*p* = 0.241) and after (*p* = 0.188) implant placement. At point 2 the highest soft tissue gain was also observed in CTG groups: 1.36 ± 0.6 (IIb) and 1.15 ± 0.5 (IIIb). The smallest increase was obtained in XCM groups: 1.0 ± 0.7 (IIa) and 0.57 ± 0.6 (IIIa). There were no significant differences in soft tissue thickness gain between the groups with XCM (IIa) and CTG (IIb) before implant placement (*p* = 0.654). But the difference between the groups with XCM (IIIa) and CTG (IIIb) 3 months after implant placement was significant (*p* = 0.042). Regardless of the time of augmentation, there was no statistically significant difference between groups using the same material [27].

In the study by Thoma et al., baseline soft tissue thickness was above 2 mm in all groups: 3.5 ± 1.0 (XCM) and 4.2 ± 1.9 (CTG) at the occlusal site (*p* = 0.442), 2.9 ± 1.5 (XCM) and 4.1 ± 2.0 (CTG) at the buccal site (*p* = 0.211), 2.6 ± 2.3 (XCM) and 3.4 ± 1.8 (CTG) at the apical site (*p* = 0.246) [15]. There were no statistically significant differences between XCM and CTG (*p* = 0.987; *p* = 0.953; *p* = 0.481) at 1 month after soft tissue augmentation. The increase in soft tissue thickness 3 months after augmentation procedure was observed mainly at the occlusal site: 1.4 ± 1.4 (XCM) and 0.8 ± 1.8 (CTG), but there were no statistically significant differences between two groups (*p* = 0.359). For all other sites, similar increases were observed: 1.1 ± 1.4 (XCM) and 0.8 ± 2.2 (CTG) at the buccal site (*p* = 0.281), 0.9 ± 1.9 (XCM) and 1.6 ± 2.6 (CTG) at the apical site (*p* = 1.000; *p* = 0.470) [15].

Zeltner et al. [28] demonstrated the results obtained using a numerical analysis of the same patient population which was reported in a previous publication by Thoma et al. [15] The median crestal regions of interest (ROI) was 24.8 mm^2^ and 23.7 mm^2^ for XCM and CTG, respectively. The corresponding values for the buccal ROI were 32.2 mm^2^ for XCM and 29.2 mm^2^ for CTG. The differences between groups were not statistically significant (crestal *p* = 0.278; buccal *p* = 0.113). The linear changes from baseline to 3 months in the crestal ROI amounted to 0.27 ± 0.26 for XCM and to 0.42 ± 0.74 for CTG. The change in the XCM group was significant (*p* = 0.002), whereas the change in the CTG group was not significant (*p* = 0.129). The differences between the two groups did not differ significantly (*p* = 0.287). The gain in soft tissue volume from baseline to 3 months in the buccal ROI was 0.77 ± 0.74 for XCM and 0.79 ± 0.45 for CTG. Changes for both XCM (*p* = 0.002) and CTG (*p* = 0.004) were significant. The differences between groups were not significant (*p* = 0.534) [28].

In the study by Baldi et al., baseline measurements of mucosal thickness were not provided [29]. The dynamics of changes in the level of the vestibular mucosa (FST) showed an increase in the first 1.5 months by 0.34 ± 0.13 (SE) in the XCM group (*p* = 0.0218) and by 0.50 ± 0.22 (SE) in the CTG group (*p* = 0.0756). The difference was statistically significant only in XCM group. After 6 months, the level of the vestibular mucosa in both groups decreased, and the difference from the initial level was 0.32 ± 0.21 (SE) and 0.35 ± 0.30 (SE) in the XCM and CTG groups, respectively. The mean differences in FST levels between group without any augmentation material and CTG group were significant for the 1.5 and 6 month follow-up, being 0.93 ± 0.76 (*p* = 0.01) and 1.32 ± 1.03 mm (*p* = 0.008). Other differences between groups were not statistically significant [29].

In the study by Cosyn et al., the initial soft tissue thickness was defined as a volume parameter of study-relevant area of interest (AOI) [26]. The mean AOI amounted to 28.63 and 28.07 mm^3^ in the control and test group, respectively. There was no significant difference between the groups (*p* = 0.553). Directly after the augmentation, a change in the vestibular contour of the soft tissues was observed. In addition, a significant time effect (within group difference) was observed in both groups. In the CTG group, the increase in buccal soft tissue profile was 1.43 mm (95% CI 1.15 – 1.70). After 3 months, the increase in buccal soft tissue profile was 1.15 mm (95% CI 0.88 – 1.43), compared to the initial level. In the XCM group, the increase in buccal soft tissue profile immediately post-surgery was 1.90 mm (95% CI 1.63 – 2.18). After 3 months, the increase in buccal soft tissue profile from baseline was 0.85 mm (95% CI 0.58 – 1.13). Three months after surgery, no significant effect of the treatment was observed, although there was a trend towards an additional increase in the buccal soft tissue profile by 0.30 mm (95% CI 0.01 to 0.61; *p* = 0.054) in favor of the control group. A significant shrinkage of CTG and XCM was defined to be 0.27 mm (95% CI 0.01 – 0.53; *p* = 0.039) and 1.05 mm (95% CI 0.79 – 1.31; *p* < 0.001). Sites treated with XCM demonstrated 0.78 mm (95% CI 0.41 – 1.14; *p* < 0.001) more shrinkage between than sites treated with CTG [26]. A 1-year follow-up study was recently published. The difference between groups in increase was 0.41 mm (98.3% CI: 0.12 – 0.69) and was significant in favor of CTG [50].

In the study by Ashurko et al., in the XCM and CTG groups, the initial mucosal thickness was comparable and amounted to 1.61 ± 0.07 and 1.63 ± 0.07, respectively [30]. After three months the mean thickness for the XCM group increased to 2.81 ± 0.11, the CTG group showed 3.16 ± 0.11. A significant gain in soft tissue thickness was observed in both groups compared to baseline. Soft tissue increasing was 1.18 ± 0.11 in the XCM group and 1.55 ± 0.11 mm in the CTG group. The difference between groups was -0.366 (− 0.66 to − 0.07; *p* = 0.016) in favor to CTG. A soft tissue thickness at least 2 mm was achieved in 93.33% (70.18 to 99.69%) of CTG group patients and 60% (35.75 to 80.18%) of XCM group (*p* = 0.08). A soft tissue thickness at least 3 mm was achieved in 46.67% (24.81 to 69.89%) of SCTG group patients and 33.33% (15.18 to 58.29%) of XCM group (*p* = 0.71) [30].

In a study by Hélio et al., the initial soft tissue thickness was 2.12 ± 0.33 for XCM group and 2.05 ± 0.33 for CTG group [31]. After three months the mean thickness for the XCM group increased to 2.61 ± 0.43, the CTG group showed 2.98 ± 0.5. A significant gain in soft tissue thickness was observed in both groups compared to baseline (for XCM *p* = 0.013; for CTG *p* < 0.001). There was no statistically significant difference between the two groups (*p* = 0.065) [31].

### Patient-Reported Outcome Measures (PROMs)

Only five [15, 25, 26, 29, 30] of the eight included studies examined patient-reported outcome measures (PROMs) (Tab 7). The intensity of post-operative pain, general discomfort and patient satisfaction with the result were recorded using a visual analogue scale (VAS). Two studies [15, 30] used the Oral Health Impact Profile Questionnaire (OHIP-G14) to assess quality of life.

In a study by Baldi et al., the patients’ mean aesthetic satisfaction was high in all three groups, with no statistically significant differences between groups found after 6 months [29]. Cairo et al. [25] found that patients tolerated surgical procedure using a collagen matrix more easily. However, there was no statistically significant difference in perceived pain between two groups. Seven days after the intervention, patients of the test group using VAS noted: lower intensity of post-operative pain, the least number of days with discomfort and less intake of anti-inflammatory drugs. After 2 weeks, the CTG group had more sites with edema. There were no statistically significant differences in other indicators in the post-operative period and after 6 months; all patients reported their high satisfaction with the result [25].

Post-operative bleeding was assessed dichotomously (yes/no) in the study by Cosyn et. al. [26] VAS was used to record the severity of post-operative pain, edema and hematoma, the number of analgesics taken and aesthetic satisfaction. In both study groups, after 7 days, no statistically significant difference was observed between the groups in terms of post-operative bleeding, pain, edema and in the use of analgesics. However, the mean score for post-operative hematoma was lower in the test group (XCM) than in the control group (CTG). Three months after the operation, there was no significant difference between the groups in the patients’ aesthetic satisfaction with the condition of the peri-implant soft tissues [26].

Thoma et al. calculated VAS scores 4 h after surgery and then daily until suture removal, as well as on days 30 and 90 [15]. When sutures were removed, the mean total scores on the OHIP-G14 questionnaire for CTG were higher than for XCM. The difference in both criteria was not statistically significant. But the results obtained for these indicators correlated with the high use of analgesics in the CTG group from the day of surgery to the day of suture removal. The median physical pain was 100% higher in the CTG group compared to the XCM group. The largest differences between groups were found for physical pain and social disability [15].

Ashurko et al. demonstrated a slightly higher VAS score for CTG between day 1 and day 7 post-surgery without being statistically significantly different at any time point (*p* > 0.05) [30]. By the 7th day, patients with XCM showed a more pronounced decrease in the quality of life (2.22 ± 0.77) compared with patients with CTG (1.87 ± 0.74); however, the difference between the groups was not statistically significant (*p* > 0.05). By the 90th day, the difference between the groups changed and amounted to 0.67 ± 0.62 and 0.73 ± 0.46, respectively (*p* > 0.05). No other significant difference was detected in the postoperative period [30].

### Peri-implant tissue health

The condition of peri-implant tissues was assessed in four [15, 25, 26, 29] of eight studies included in this systematic review. The condition of peri-implant was analyzed according to the following indicators: bone level, probing depth, bleeding on probing, plaque, clinical attachment level.

In the study by Cairo et al., during 6 months after intervention, there was no statistically significant difference in mean bone level between groups (0.1 mm difference; 95% Cl – 0.1 to 0.3; *p* = 0.022) [25]. There was also no significant difference in other parameters between the groups [25].

In Cosyn et al. study, the probing depth and bleeding on probing were assessed after 3 months after implantation [26]. Plaque and bleeding on probing were between 20 and 30% after 3 months and did not vary between groups, however the difference in probing depth between groups was 0.30 mm (95% CI 0.06 – 0.54; *p* = 0.017) and was statistically significant in favor of control group (CTG). Mean marginal bone loss was 0.34 mm in control group (CTG) and 0.72 in test group (XCM) after 3 months. The difference was 0.38 mm (95% CI 0.15—0.60; *p* = 0.001) and was statistically significant in favor of control group [26]. In the study by Thoma et al., clinical and periodontal measurements were assessed before surgery, 30 and 90 days after the intervention. As a result, there was no significant difference between groups considering all measurements [15]. Baldi et al. assessed only bone level around implant after 6 weeks after soft tissue augmentation and at the time of permanent crowns installation. There was no statistically significant difference between groups [29].

### Width of keratinized Mucosa

The width of keratinized mucosa (KMW) was evaluated in five [15, 25, 29–31] of the eight included studies. In a study by Cairo et al. at the 6-month follow-up visit surgery resulted in a significant increase in KMW (1.1 and 0.9 mm for XCM and CTG respectively). The difference between groups was not statistically significant [25]. In study by Baldi et al. there was not statistically difference between XCM and CTG, and both treatments demonstrated a significant increase (1.05 and 0.80 mm respectively) from baseline to six-month follow-up [29].

In study by Thoma et al. there was no statistically significant difference in KMW between groups at the mesial neighboring tooth (*p* = 0.264) and implantation site (*p* = 0.624). At the distal neighboring tooth the difference in KMW between groups was statistically significant (1.1 mm; Q1: 2.0; Q3: 0.1) (*p* = 0.029) [15].

In study by Ashurko et al. the amount of KMW was not changed in both procedures 3 months after intervention and the similar final amount of KMW was observed with no significant difference [30].

Hélio et al. showed increasing of KMW from 3.00 ± 0.65 to 3.67 ± 0.69 (*p* = 0.003) for XCM and from 3.21 ± 0.71 to 4.41 ± 0.55 (*p* < 0.001) for CTG [31]. When comparing the 2 types of grafts in study, it was observed that the final KMW was higher in CTG group, with statistically significant difference between the two types of grafts (*p* < 0.014) [31].

### Esthetic Outcomes

Three [25, 26, 29] of eight included studies evaluated aesthetic outcomes. The Cosyn et al. assessed mid-facial recession (MFR), pink aesthetic score (PES) and mucosal scarring index (MSI) [26]. Mid-facial recession was calculated 3 months after soft tissue augmentation by subtracting the level of mid-facial soft tissues (the distance from the incisal edge of the crown to the buccal edge of the mucosa in the center of the implant) from the postoperative level of mid-facial soft tissues. Positive values indicated the onset of a recession, while negative values indicated a vertical regrowth. PES and MSI were assessed at 3 months from occlusal and anterior photographs by scores: pink aesthetic score from 0 to 14 (worst to perfect aesthetic result), and mucosal scarring index from 0 to 10 (from no scar to worst). Mid-facial recession was significantly higher in XCM group than in CTG group. The authors attributed this result to the large thickness of XCM, which could provoke a more coronal location of the flap in the test group. In addition, it was concluded that there was no clinical significance in more pronounced mid-facial recession in the control group, as there was no significant difference between PES in the control and test groups. The MSI was low in both groups, as evidenced by the sufficient peri-implant esthetic outcome in both groups [26].

In a study by Baldi et al., aesthetics was evaluated both by the operating physician and by the patient at the stage of permanent crown installation [29]. The surgeon assessed aesthetics using a pink-aesthetic score, which included evaluation of each variable: medial and distal papilla, soft tissue level, alveolar ridge deficiency, and soft tissue color and texture. Patients also expressed their degree of satisfaction with implant treatment in general, answering questions from the questionnaire using a visual analogue scale. As a result, there were no statistically significant differences between the three study groups in PES, but there was a significant difference in interdental papilla index between CTG group and no graft group. Mean esthetic patient satisfaction was high in all groups, and no statistical differences were found between them [29].

Cairo et al. collected outcomes from patients regarding aesthetics (soft tissue and crown appearance) and results of VAS [25]. It was found that after 6 months (at the last appointment), patients were highly satisfied with the aesthetic results with no significant difference between the groups [25].

### Histological findings

In this systematic review, among the included studies only three [15, 30, 31] reported histological findings.

Thoma et al. evaluated obtained biopsies 90 days after grafting XCM (volume stable collagen matrix) and CTG histologically to study the amounts of remaining XCM and new connective tissue formations [15]. In both groups’ vascularization was observed throughout the specimens with limited amount of inflammatory cells. In the CTG group biopsies there was a relatively loose network of collagen fibers with and no differentiation between grafted and newly formed connective tissue. A dense collagen fiber network as well as identification of remaining XMC with the remodeling processes was observed in the XCM group. The histomorphometric assessment showed 32.1% (± 18.5%) of a remaining matrix body, and 30.1% (± 11.8%) mean amount of connective tissue in XCM group, while in the group with CTG the mean amount of newly formed and grafted connective tissue reached 77.6% (± 11.6%) [15].

In the study by Ashurko et al., it was revealed that in both groups, after 3 months the newly formed mucous membrane of biopsy specimens was lined with stratified squamous epithelium of various thicknesses, which was delimited from the papillary layer by a basement membrane [30]. The indicators of the average and maximum thickness of the epithelial layer were less than in CTG group than in XCM group. There were no differences in the true average thickness (relative area) of the layers in the two groups. The CTG group had significantly longer rete ridges. At the same time, this index did not significantly affect the length of the basal membrane, although its relative length was slightly longer than in the XCM group. The indicators of the proliferative capacity of the epithelium, represented by the relative cellularity of the basal layer and the proportion of mitoses in the basal layer in the XCM group were also significantly lower than in the CTG group [30].

Hélio et al. found that 3 months after soft tissue augmentation XCM and CTG did not present a statistically significant difference in the number of fibroblasts per area close to the epithelium (with no grafted material present) and an area close to the periosteum (within the graft area) with no clinical and histological signs of inflammatory process [31].

### Complications

All included studies were analyzed for reported complications. Cosyn et al. reported complications in the CTG group: intolerable pain and edema, removal of the implant due to mobility after 1 week, wound dehiscence after 1 week, and in the XCM group: severe post-operative bleeding and wound dehiscence after 1 week [26]. Cairo et al. described 1 mm soft tissue recession 6 months post-op [25]. Ashurko et al. described complicated healing in 2 patients of the XCM group [30]. At the time of suture removal (day 14), the discrepancy of the wound edges with exposure of the granulating surface of the collagen matrix was determined. In both cases, the patients did not notice any discomfort during healing, no exposure of the implant cap screws was detected, and the wound healed by secondary intention [30].

## Discussion

### Summary of main results

The present systematic review and meta-analysis addresses the question: “Can modern XCM provide results comparable to autogenous CTG in increasing soft tissue thickness around dental implants?” Furthermore, such parameters as KMW, PROMs, aesthetic outcomes, peri-implant health, and histological findings have been analyzed.

Based on our systematic review and meta-analysis, it can be argued that the use of XCM, as well as CTG, leads to an increase in the thickness of soft tissues in the implant area. Measurement of soft tissue changes after augmentation procedures is associated with certain difficulties, primarily because the changes are shallow and sometimes hard to detect. The standard or analog measurement method has been used in most studies (periodontal probe, endodontic probe, etc.), [18, 31, 34, 36] although recently, more modern methods using digital technologies, [26, 28, 30, 35] and ultrasonic devices [27] find their application. Still, in the most of the studies analog techniques were utilized, which have their drawbacks compared to more modern digital methods.

Baldi et al. used an analog method to measure changes in mucosal thickness along a reference line, which characterizes changes in soft tissue contour in height [29]. The article does not describe the measurement methodology itself in sufficient detail: in which part of the ridge the measurement was made, how the reproducibility of the measurement at the same point was controlled, with what accuracy the measurement was made and what instrument was used. There might have been some bias in the study outcomes. For the same reason, the author has no data on the initial thickness of the soft tissues since the used technique was non-invasive.

A more classic technique with an injection needle was used in a study by Cairo et al. [25]. The main disadvantage of this method is that the data obtained refer to only one specific point of the dental implant site. In addition, it is difficult to determine the same point at different periods of time, which may produce bias. The same problems should be noted in the study by Hélio et al. [31].

Fabrication of a customized stent with openings on the occlusal and buccal sides ensures reproducibility of gathered measurements, as demonstrated in the study by of Thoma et al. [15]. Furthermore, such approach makes it possible to receive data from several points providing a better overview of tissue reaction.

The utilization of digital technologies allows performing a volumetric assessment based on the comparison of digital models received in different time periods. This method allows obtaining data regarding the volume changes of the entire augmentation area [35]. A similar approach is described in the studies by Zeltner et al. and Ashurko et. al. [28, 30]. Despite the fact that Zeltner et al. [28] conducted measurements on a group of Thoma et al. [15], we decided to include it in this review, since the author used a more modern, digital version of the measurement of volumetric changes after augmentation and obtained completely new results. This once again tells us about the difficulty of comparing.

Digital method is non-invasive and does not cause radiation exposure. A disputable issue in the study by Zeltner et al. and Ashurko et al. is that taking analog impressions may produce some inaccuracies of the entire analysis. This is especially true for areas with mucosal reflections [28, 30]. The utilization of an intraoral scanner may overcome this problem, as demonstrated by Cosyn et al. [26]. As the author of the publication noted, the main drawback of this technique is that the area of interest in all patients was different and varied depending on individual differences in anatomical structures [26]. This problem was solved in a special way in the study by Célien Eeckhout et al., which was not included in this review. In order to make a direct comparison between patients with areas of interest differed in size, the researchers converted the mean volume change per area into a mean linear change in buccal soft tissues profile in mm [35]. Another problem in the study by Cosyn et al. is that restorations were installed at the same time with soft tissue grafting [26]. It is related to the limitation of this method, since the technique of comparing 3D models makes it possible to adequately compare only equivalent areas, without any superstructures. After placing the crown, the soft tissues are usually displaced, which can create the illusion of an increase in volume of the tissues. No randomized trials have been found to confirm or reject this assumption and this is a subject for further investigation.

The use of an ultrasonic device to measure soft tissue thickness is also of interest. This technique was used in a study by Puzio et al., but there is a problem with reproducibility of the reference points in the study due to the availability of only conditional landmarks [27].

In the present study, an attempt has been made to analyse the data on mucosal thickness gain in the area of dental implants depending on the thickness assessment method used: digital or analogue. However, since there were few studies that fits the inclusion and exclusion criteria, we had to summarise the data of buccal and occlusal measurements for the analysis. Based on the meta-analysis we can cautiously conclude that, there is no statistically significant difference between the methods used. However, more studies with subgrouping are needed for a definitive understanding.

According to the meta-analysis, soft tissue growth occurred more on the vestibular surface than on the occlusal one. Zeltner et al. assumes that this may be due to an increased pressure on the transplants in the crestal region caused by the primary wound closure and sutures position. XCM has high elasticity, and it is not as resistant to mechanical load as CTG. Possibly for this reason, there was a more pronounced difference in volume between the buccal and crestal regions of interest in the XCM group [28]. In addition, deficiency in the occlusal site may be due to more frequent soft tissue dehiscence defects after suture removal (30% (VCMX) and 10% (SCTG)) and healing by secondary intention [15].

In most of the reviewed studies, the long-term follow-up was 3 months [15, 19, 26, 28, 30, 31]. It is known, that volume decrease may be associated with remodeling during the initial phase of wound healing, which is most pronounced in the first three months after soft tissue augmentation [34, 51]. However, only minimal changes are observed between 3 and 6 months [16, 52]. Nevertheless, according to some researchers, 6 months is the optimal time to assess the effectiveness of soft tissue augmentation. Because the collagen matrix is completely degraded at this time, that allows a more objective assessment of the result [36, 44, 53].

In high number of RCT, which were included in this systematic review, standard periodontal tests were assessed: bone level, probing depth, bleeding on probing, plaque, clinical attachment level. In should be noted that the majority of clinicians agree on the lack of statistically significant difference in these tests between groups [15, 18, 25, 29, 48].

Most researchers also agree that sufficient width of the keratinized attached mucosa reduces the risk of plaque accumulation, the occurrence of mucosal recession and peri-implantitis, and plays an important role in maintaining peri-implant health [54, 55]. Therefore, in studies, in addition to the actual increase in the thickness of the peri-implant soft tissues, it is also necessary to pay attention to the increase in the width of the efficiency factor. Analyzing the results of the included studies, it can be noted that most clinicians reveal a significant increase in KMW using collagen matrix and autogenous graft, but do not indicate statistically significant differences in this parameter between groups [15, 25, 29]. In recent years, several recent systematic reviews and meta-analyses also did not reveal a statistically significant difference in KMW gain between the use of XCM and CTG [17, 21].

The studies of most authors report a more comfortable postoperative period with the use of soft tissue substitutes, which is accompanied by less overall pain and less consumption of painkillers compared with the use of CTGs [15, 18, 25, 46, 56]. However, it should be noted that the severity of postoperative discomfort and pain depends on the localization of the donor area and the method of harvesting CTG. According to some studies, the absence of morbidity in the donor area is due to more comfortable healing after CTG harvesting from the region of the maxillary tuberosity, compared to the area of the hard palate [57]. Despite the difference in the methods of assessing aesthetic results, in most studies, patients were highly satisfied with the results of treatment, which is confirmed by the questionnaire data given above.

An adequate soft tissue volume around dental implants is an important component in achieving not only the protection from mechanical damage and the occurrence of inflammatory complications, but also to obtain a higher aesthetic result of implant treatment [42, 58]. In this regard, aesthetics assessment is included in the secondary outcomes of some studies focusing on peri-implant soft tissue augmentation. Despite the difference in methods for assessing aesthetic outcomes, in most studies, [25, 26, 29] patients who underwent soft tissue augmentation were highly satisfied with the results of the treatment. Objective methods for assessing aesthetic outcomes (PES, MSI, MFR) also showed no statistically significant differences between CTG and XCM groups [25, 26, 29].

It should be mentioned, that it is important to assess the outcomes in the long term, as was done by Thoma et al., who evaluated results 3 years after installation of permanent restorations and found minor differences and stable results in terms of buccal contour, marginal bone level and aesthetics [47].

### Agreements and disagreements with other reviews

A systematic review by Gargallo-Albiol et al. published in 2019 found no significant difference between the use of XCM and CTG to increase soft tissue thickness [17]. In contrast, the present study showed a higher effectiveness of CTG compared to XCM. It can be assumed, that this discrepancy may be due to several reasons. First, Gargallo-Albiol al. reviewed included RCTs with a different surgical approaches (bilaminar technique and apically positioned flap), which could contribute to some heterogeneity. Second, 4 RCTs [26, 29–31] included in this systematic review were published after the Gargallo-Albiol et al. study.

Despite this, the results of this review correspond to the results of most of the previous studies [12, 21, 59, 60]. It is worth noting that the authors of most reviews included not only RCT, but also CCT, which may have influenced the results of the study. In addition, in some studies, the authors compared the soft tissue thickness gain after various surgical techniques (bilaminar technique, apically positioned flap, using of a free gingival graft, etc.), which can be compared with each other only by using a network meta-analysis [12]. A recently published systematic review by Valles et al. found a statistically significant difference in the increase of soft tissue thickness in the group using CTG compared to soft tissue substitutes. It should be noted that the authors included not only collagen matrix, but also ADM, PRF, T-PRF in the group of soft tissue substitutes, which diverges from the objectives of our review. It should also be mentioned that during the period indicated by the authors, there were several RCTs [28, 29, 31] matching the inclusion criteria that were not added to the systematic review [59]. In order to expand the available evidence, we have added more recent studies, allowing us to conduct a review and meta-analysis of the maximum number of RCTs to date.

### Quality of evidence

The certainty of evidence from the most important outcomes of this study was also analyzed. The GRADE assessment is an essential point in determining the methodological quality of the articles included in the systematic review. The GRADE approach includes five domains for rating down certainty (risk of bias, inconsistency, indirectness, imprecision and publication bias) [61]. The results obtained from the GRADE criteria-based assessment of the studied outcomes showed that the articles included in this systematic review and meta-analysis had a moderate or high certainty level. The decrease in the confidence level for three of the five outcomes from high to moderate was influenced by a high risk of bias in one of the included studies [31].

### Limitations

This systematic review presents some limitations that should be considered.

First, during the study we conducted literature search in the following databases: PubMed (MEDLINE), Scopus, Cochrane Library, LILACS, eLIBRARY.RU. Although other databases are known to exist (e.g. Embase and Web of Science). Thus it can be assumed that there are more scientific publications that were not included in this study, although this was compensated for by the manual search that was conducted. Unpublished studies, the gray literature, nonprofit reports, government studies and other materials were also reviewed electronically using EASY search.

Second, few studies with different design that involved recording changes in soft tissue thickness by different methods and at different control points were included.

Third, the analysis cannot be considered completely objective due to the fact that in some studies soft tissue augmentation was accompanied by full wound closure, and in another part of the studies healing abutment was immediately installed, which could potentially affect the reliability of the results.

The small number of included studies does not allow to provide an objective assessment of the amount of soft tissue gain from the occlusal surface, because this parameter was analysed only in 3 studies. Moreover, the same patient population was analysed using different methods in the Thoma and Zeltner studies [15, 28]. Furthermore, when performing subgroup meta-analyses and attempting to compare digital and analogue measurement methods, we encountered an even greater reduction in the power of subgroup comparison. Therefore, we decided to combine the results of measurements and subsequent analyses of the vestibular and occlusal subgroups, which also represents a limitation of this paper.

In our study we conclude that XCM is less effective than CTG in increasing soft tissue thickness around dental implants. However, our statement must be interpreted with caution given the limited number of articles included in the meta-analysis and high variability in the outcome measures in the studies. Consequently, there is a requirement for further long-term researches.

## Conclusion

Within the limitations associated with the insufficient number of studies analysed in this study, the present systematic review and meta-analysis suggest that XCM is less effective than CTG in increasing soft tissue thickness around dental implants. However, XCM also provides soft tissue thickness gain and can be recommended for use in various clinical situations.

## Data Availability

The data is available on reasonable request.
